# Specific intracellular retention of circSKA3 promotes colorectal cancer metastasis by attenuating ubiquitination and degradation of SLUG

**DOI:** 10.1038/s41419-023-06279-w

**Published:** 2023-11-16

**Authors:** Jingwen Deng, Shaoxia Liao, Chaoyi Chen, Fengyan Han, Siqin Lei, Xuan Lai, Kehong Ye, Qizheng Han, Fang E, Chao Lu, Maode Lai, Fanlong Liu, Honghe Zhang

**Affiliations:** 1 Department of Pathology and Women’s Hospital, Zhejiang University School of Medicine, Research Unit of Intelligence Classification of Tumor Pathology and Precision Therapy, Chinese Academy of Medical Sciences (2019RU042), 310058 Hangzhou, China; 2 Department of Colorectal Surgery and Oncology, Key Laboratory of Cancer Prevention and Intervention, Ministry of Education, The Second Affiliated Hospital, Zhejiang University School of Medicine, Hangzhou, Zhejiang China; 3 Department of Chemistry, University of South Florida, Tampa, FL 33620 USA; 4 Department of Pathology, Research Unit of Intelligence Classification of Tumor Pathology and Precision Therapy of Chinese Academy of Medical Sciences (2019RU042), Zhejiang University School of Medicine, 310058 Hangzhou, China; 5 Key Laboratory of Disease Proteomics of Zhejiang Province, Zhejiang University, Hangzhou, 310058 China; 6 Department of Colorectal Surgery, The First Affiliated Hospital, College of Medicine, Zhejiang University, 310058 Hangzhou, China

**Keywords:** Colorectal cancer, RNA, RNA splicing

## Abstract

Our previous study demonstrated that tumor-suppressor circular RNAs (circRNAs) can be specifically secreted outside of colorectal cancer (CRC) cells within exosomes to maintain tumor cell fitness. However, whether tumor-driving circRNAs can be specifically retained in cells to facilitate tumor progression remains unknown. In this study, circRNA-seq showed that circSKA3 was significantly upregulated in CRC tissues but downregulated in serum samples from CRC patients. In addition, circSKA3 promoted CRC progression in vitro and in vivo and was retained in CRC cells via a specific cellmotif element. Interestingly, the cellmotif element was also the site of interaction of circSKA3 with SLUG, which inhibited SLUG ubiquitination degradation and promoted CRC epithelial–mesenchymal transition (EMT). Moreover, FUS was identified as a key circularization regulator of circSKA3 that bound to the key element. Finally, we designed and synthesized specific antisense oligonucleotides (ASOs) targeting circularization and cellmotif elements, which repressed circSKA3 expression, abolished the SLUG–circSKA3 interaction, and further inhibited CRC EMT and metastasis in vitro and in vivo.

## Introduction

Recently, circular RNAs (circRNAs) have been considered important epigenetic regulators that participate in various tumor biological processes, such as cell proliferation, apoptosis, autophagy, angiogenesis, energy metabolism, immune surveillance, migration, and invasion [1]. Generally, circRNAs are produced by back-splicing following parent gene transcription. CircRNAs play biological roles by sponging miRNAs, binding to functional RNA-binding proteins (RBPs), forming pseudogenes after reverse transcription, regulating gene transcription or RNA splicing in the nucleus, and even encoding polypeptides [2].

To date, many specific circRNAs have been identified in multiple types of tumors that regulate tumorigenesis and progression; for example, circVAMP3 accelerates the formation of stress granules and suppresses the translation of the proto-oncogene c-myc in hepatocellular carcinoma (HCC) [3], and circANAPC7 can inhibit pancreatic cancer progression via the PHLPP2-AKT-TGF-β signaling axis [4]. Through analysis of circRNA-sequencing data, we previously found that some circRNAs are downregulated in tumor tissue samples but upregulated in serum samples. We further investigated this interesting result and found that tumor cells can actively excrete the tumor-suppressive circRNA circRHOBTB3 in exosomes to sustain cancer cell fitness. This mechanism causes a low level of circRHOBTB3 in tumor tissue and a high level of circRHOBTB3 in serum in colorectal cancer (CRC) patients [5]. Nevertheless, it remains unknown why some other circRNAs are upregulated in tumor tissue samples but downregulated in serum samples.

CircSKA3 (hsa_circ_0000467) is formed by circularization of exon 4 of the spindle and centromere-associated protein 3 (SKA3) gene, of which expression was just increased in CRC tissues but decreased in serum samples. The parent gene SKA3 is located on human chromosome 13q and regulates cell mitosis, proliferation, apoptosis, and eukaryote development [6, 7] by forming a heterodimer with SKA1 or SKA2 and participating in the formation of the spindle and centromere [8]. Previous studies have found that abnormally high expression of SKA3 is closely related to the occurrence and development of HCC [9], laryngeal squamous cell carcinoma [10], and breast cancer [11]. In addition, several studies have reported that circSKA3 expression is dysregulated in breast cancer [12] and medulloblastoma [13]. However, the clinical significance and biological role of circSKA3 in CRC have not yet been reported. In this study, we investigated the biological role and molecular mechanism of circSKA3 in CRC metastasis and explored the regulation of circSKA3 retention inside CRC cells. Furthermore, specific antisense oligonucleotides (ASOs) targeting circSKA3 were designed and synthesized to intervene in CRC metastasis, which could become novel therapeutic agents for cancer in the future.

## Results

### CircSKA3 levels are increased in CRC tissues but decreased in serum exosomes from CRC patients

**Fig. 1:**
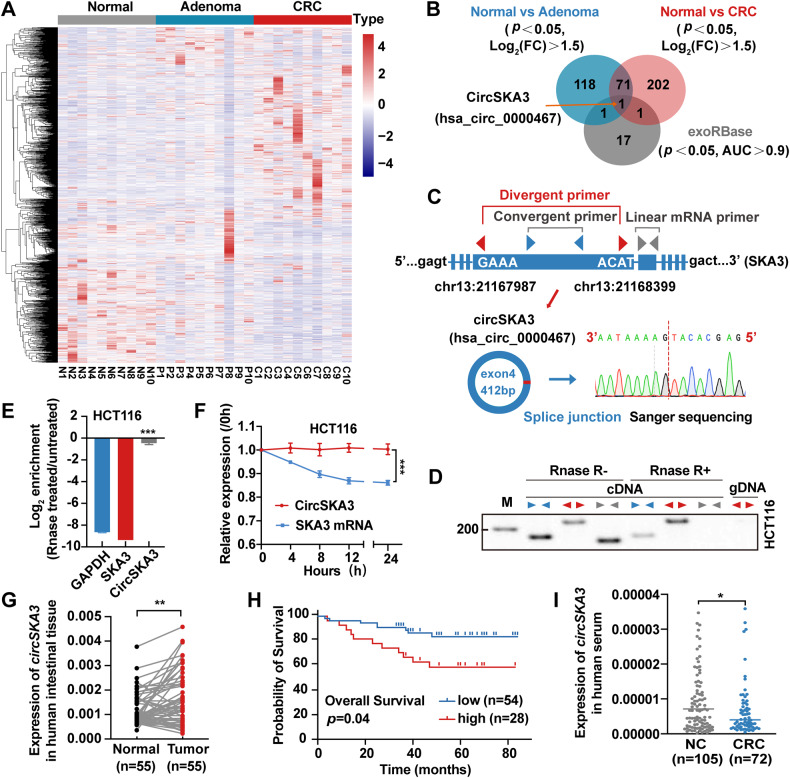
CircSKA3 characteristics and expression in CRC. **A** CircRNA-sequencing heatmap of 10 paired normal intestinal mucosa–adenoma/adenocarcinoma tissue samples. **B** Venn diagram of upregulated circRNAs in CRC, adenoma and serum exosomes from CRC patients in the GEO database with AUC > 0.9. **E** Schematic illustration of circSKA3 circularization from exon 4 of the SKA3 gene and Sanger sequencing of the BSJ site of circSKA3. **C**, **D** The head-to-tail splicing of circSKA3 was clarified in cDNA and gDNA of HCT116 cells by RT‒PCR with different primers and RNase R treatment. **E** The stability of circSKA3 and linear SKA3 in HCT116 was assessed by RNase R treatment followed by RT‒qPCR. **F** Evaluation of the stability of circSKA3 and SKA3 mRNA in HCT116 treated with actinomycin D. **G** Expression levels of circSKA3 in 55 paired normal and CRC samples as determined by RT‒qPCR (normalized to GAPDH). **H** K–M survival analysis of overall survival in CRC patients according to the circSKA3 expression level. **I** Detection of circSKA3 in the serum of 105 normal controls and 72 CRC patients. All results are representative of three independent experiments, and shown as mean ± SD. Statistical significance was accessed by paired-samples Student’s *t*-tests (**G**), Student’s *t*-test (**E**, **F**, **I**), and log-rank test (**H**). *p < 0.05, **p < 0.01, ***p < 0.001.

First, we performed circRNA sequencing on 10 paired normal mucosa samples, adenoma and adenocarcinoma tissue samples from 10 CRC patients, and we identified 191 upregulated circRNAs in adenoma samples and 277 upregulated circRNAs in adenocarcinoma samples compared with normal tissues (Fig. 1A, B). Then, we reanalyzed the GSE100206 and GSE100063 GEO datasets, which included serum exosomal circRNA-sequencing data from 32 healthy donors and 12 CRC patients. The results showed that the levels of 20 circRNAs were significantly changed in CRC patient serum with an area under the receiver operating characteristic (ROC) curve (AUC) > 0.9 (Fig. S1A and S1B). However, only hsa_circ_0000467 was identified as common between the differentially expressed circRNAs in adenoma and adenocarcinoma tissues, and the significantly changed circRNAs in CRC patient serum (Fig. 1B). Unexpectedly, hsa_circ_0000467 expression was significantly increased in colorectal adenoma and adenocarcinoma tissues (Fig. S1C) but decreased in CRC patient serum (Fig. S1D), with an AUC of 0.9674 (Fig. S1E).

Because hsa_circ_0000467 is produced by splicing and circularization of exon 4 of the host gene SKA3, which is located on human chromosome 13, we hereafter refer to it as circSKA3. To define the existence and circularization characteristics of circSKA3, we designed specific divergent primers for its back-splicing junction (BSJ) site to detect the expression of circSKA3, a specific linear primer outside exon 4 to detect its host gene SKA3, and convergent primers that could simultaneously detect the expression of circSKA3 and SKA3. Then, the specific BSJ site of circSKA3 was verified by RT‒PCR and Sanger sequencing (Fig. 1C). As shown in Fig. 1D, the divergent primers were able to specifically detect circSKA3 in RNase R-treated or untreated samples, but the linear primer detected the host gene SKA3 only in RNase R-untreated samples, not in treated samples (Fig. 1D). Moreover, RT‒qPCR confirmed that circSKA3 resisted RNase R digestion (Fig. 1E and S1F), and circSKA3 was more stable than linear SKA3 after actinomycin D treatment (Fig. 1F and S1G). Therefore, we used divergent primers to detect the expression of circSKA3 in CRC tissue samples and found that circSKA3 was upregulated in tumor samples compared with paired normal samples (Fig. 1G). Kaplan‒Meier (K–M) survival analysis of overall survival showed that a high level of circSKA3 was significantly correlated with poor prognosis in CRC patients (Fig. 1H). However, the level of circSKA3 was significantly decreased in the serum of CRC patients compared with that of normal controls (Fig. 1I). Taken together, these clinical data from tumor tissues demonstrate that circSKA3 might play an oncogenic role in CRC.

### CircSKA3 promotes CRC cell migration, invasion, and metastasis

**Fig. 2:**
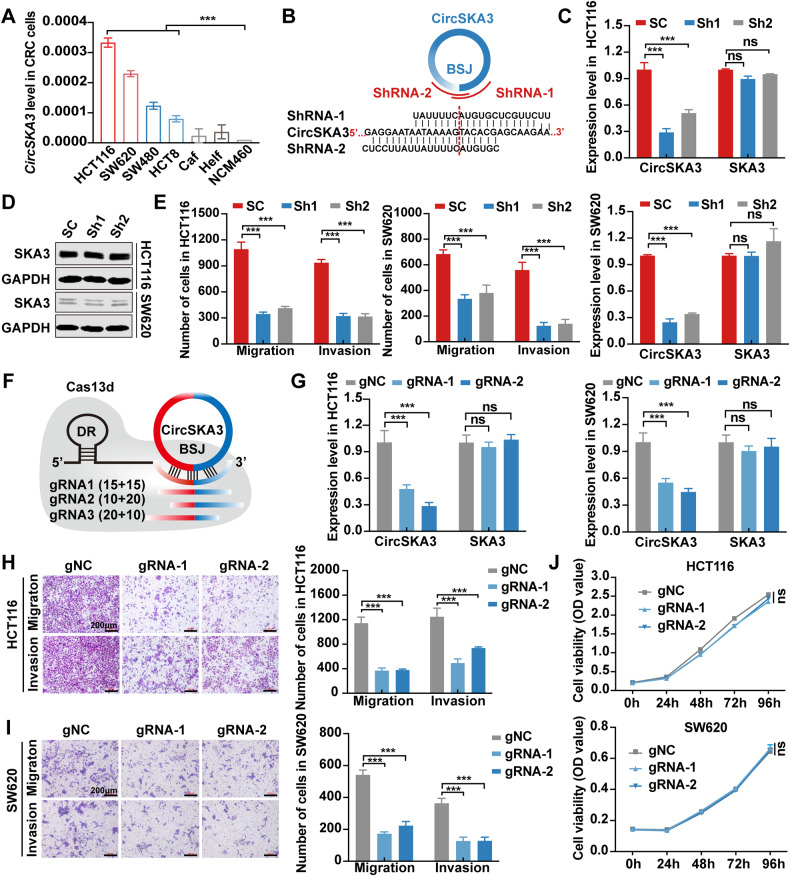
CircSKA3 promotes CRC migration and invasion in vitro. **A** Expression of circSKA3 in colorectal cell lines. **B** Schematic design of the shRNA sequence targeting the BSJ site of circSKA3. **C** Knockdown of circSKA3 via shRNA in HCT116 and SW620 cells. **D** Detection of the protein level of the host gene SKA3 after circSKA3 knockdown by immunoblotting. **E** Quantitative analysis of the migration and invasion abilities of HCT116 and SW620 cells after circSKA3 knockdown by Transwell assay. **F** Schematic of sgRNA design for the circSKA3 BSJ site. **G** Efficiency of circSKA3 knockdown by RfxCas13d-BSJ-gRNA in HCT116 and SW620 cells. **H**, **I** Transwell assay for the migration and invasion abilities of HCT116 and SW620 cells after circSKA3 knockdown by RfxCas13d-BSJ-gRNA. The right graph shows the quantitative analysis results (scale bar = 200 μm). **J** Effects of circSKA3-specific knockdown by RfxCas13d-BSJ-gRNA on HCT116 and SW620 cell proliferation. All results are representative of three independent experiments, and shown as mean ± SD (**C**, **G**, **J**) or mean ± SEM (**A**, **E**, **H**, **I**). Statistical significance was accessed by one-way ANOVA (**A**, **C**, **E**, **G**, **H**, **I**, **J**). ns, not significant with *p* > 0.05, **p < 0.01, ***p < 0.001.

To elucidate the biological roles of circSKA3 in CRC, we detected the expression of circSKA3 in CRC cell lines. The circSKA3 levels in CRC cell lines (HCT116, SW620, SW480, and HCT8) were higher than those in the other cell types, including NCM460, Helf, and Caf (Fig. 2A). Therefore, we knocked down circSKA3 in HCT116 and SW620 cells using two siRNAs to target the BSJ site of circSKA3 (Fig. S2A) and found that these siRNAs significantly silenced circSKA3 but did not affect the host gene SKA3 (Fig. S2B). CircSKA3 knockdown also significantly inhibited the migration and invasion of HCT116 (Fig. S2C) and SW620 cells (Fig. S2D). Next, we used shRNAs to construct cell lines with stable circSKA3 knockdown (Fig. 2B) and confirmed that the shRNAs specifically knocked down circSKA3 without affecting the host gene SKA3 at the mRNA level (Fig. 2C) or protein level (Fig. 2D). Knockdown of circSKA3 via shRNA also significantly inhibited migration and invasion (Figs. S2E and 2E) but only mildly inhibited cell proliferation (Fig. S2F). To further confirm the oncogenic role of circSKA3 in CRC, we knocked down circSKA3 using CRISPR-RfxCas13d/BSJ-gRNA, which specifically targeted the circSKA3 BSJ site in HCT116 and SW620 cells (Fig. 2F). When circSKA3 was specifically silenced in HCT116 and SW620 cells (Fig. 2G), migration and invasion were significantly suppressed (Fig. 2H, I), but cell proliferation was not significantly changed (Fig. 2J).

**Fig. 3:**
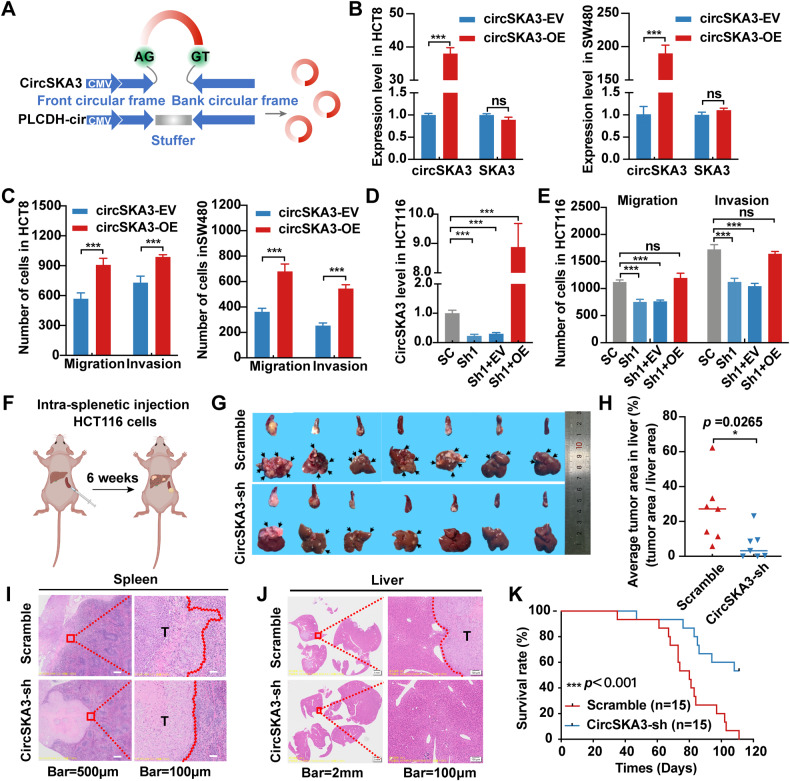
CircSKA3 promotes CRC metastasis in vitro and in vivo. **A** Schematic of circSKA3 overexpression plasmid construction based on the pLCDH-ciR vector. **B** Overexpression of circSKA3 via lentiviral expression vector in HCT8 and SW480 cells. **C** Quantitative analysis of the migration and invasion abilities of HCT8 and SW480 cells after circSKA3 overexpression by Transwell assay. **D** Overexpression of circSKA3 in circSKA3-knockdown HCT116 cells. **E** Quantitative analysis of the migration and invasion abilities of circSKA3-knockdown HCT116 cells after re-expression of circSKA3 by Transwell assay. **F** Schematic diagram of mouse spleen-liver metastasis model construction in nude mice. **G** Images of nude mouse spleens and livers. The arrows indicate metastases (*n* = 7). **H** Quantitative analysis of the average tumor area in the distant liver metastasis model. **I** H&E staining of the spleen in situ tumors in nude mice. **J** H&E staining of liver metastatic foci. **K** Survival analysis of nude mice with distant liver metastases. All results are representative of three independent experiments, and shown as mean ± SD (**B**, **D**, **H**, **K**) or mean ± SEM (**C**, **E**). Statistical significance was accessed by Student’s *t*-test (**B**, **C**, **H**), one-way ANOVA (**D**, **E**), and log-rank test (**K**). ns, not significant with *p* > 0.05, *p < 0.05, ***p < 0.001.

Furthermore, we constructed a circSKA3 overexpression vector (Fig. 3A) and used it to transfect HCT8 and SW480 cell lines. Overexpression of circSKA3 did not change the expression of the host gene SKA3 at the mRNA (Fig. 3B) or protein level (Fig. S3A). In addition, as expected, circSKA3 overexpression significantly promoted migration and invasion (Fig. 3C and S3B) but did not change the proliferation potential (Fig. S3C) in CRC cells. When we re-expressed circSKA3 in HCT116 cells with shRNA-mediated circSKA3 knockdown (Fig. 3D), the migration and invasion potential was rescued (Figs. 3E and S3D). These data demonstrate that circSKA3 can promote CRC cell migration and invasion in vitro.

To further evaluate the biological effect of circSKA3 on CRC progression in vivo, HCT116 cells stably transfected with circSKA3-shRNA and its control plasmid (Scramble) were injected into the spleens of BALB/c nude mice in situ to construct a liver metastasis model (Fig. 3F). Consistent with the in vitro results, the number (Figs. 3G and S3E) and tumor area (Fig. 3H) of liver metastatic foci were significantly lower in the circSKA3-shRNA group than in the control group. Moreover, H&E staining not only revealed a less malignant, invasive, irregular edge in the spleen (Fig. 3I) but also confirmed that the number and volume of liver metastases were reduced in the circSKA3-shRNA group of mice (Fig. 3J). Survival analysis of mice with splenic orthotopic liver metastasis showed that the knockdown of circSKA3 significantly improved the prognosis (Fig. 3K). Taken together, these results clarify that circSKA3 plays an oncogenic role in the progression of CRC independent of its host gene SKA3.

### Oncogenic circSKA3 was retained in CRC cell cytoplasm

**Fig. 4:**
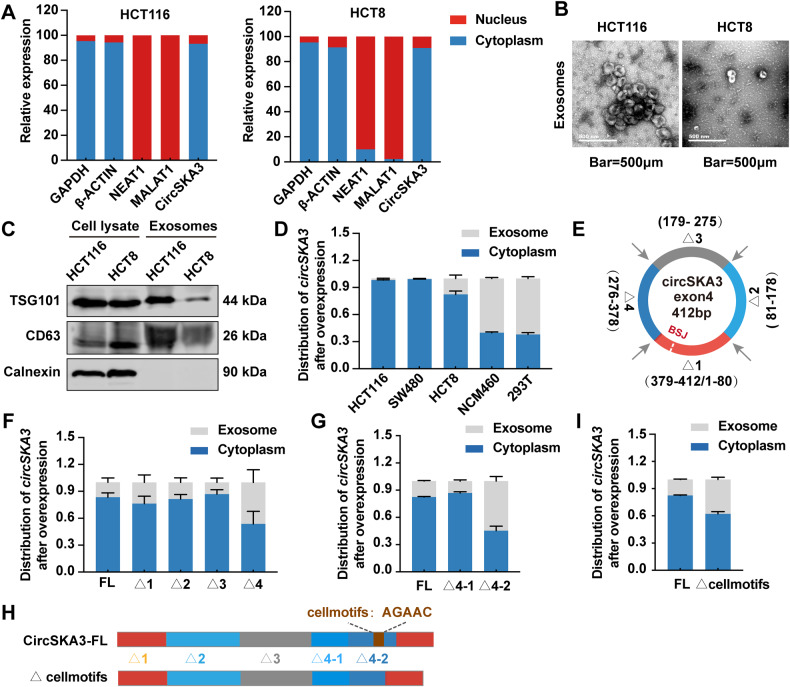
CircSKA3 retention in CRC cell cytoplasm. **A** Detection of the distribution of circSKA3 in HCT116 and HCT8 cells by RT‒qPCR. **B**, **C** Identification of exosomes in HCT116 and HCT8 cells using transmission electron microscopy and immunoblotting with the exosome-associated proteins TSG101 and CD63 (calnexin and cell lysates were used as a negative control and positive loading control, respectively) (scale bar = 500 μm). **D** Exosome secretion assay for CRC cells, NCM460 normal intestinal epithelial cells and 293T cells transfected with the circSKA3 overexpression vector. **E** Schematic diagram for circSKA3 truncation plasmid construction. **F** Exosome secretion assay for HCT8 cells transfected with truncated circSKA3 overexpression vectors (Δ1, Δ2, Δ3 and Δ4). **G** Exosome secretion assay for HCT8 cells transfected with truncated circSKA3 overexpression vectors (Δ4-1 and Δ4-2). **H** Schematic of the circSKA3 FL and cellmotif (AGAAC) truncation constructs. **I** Exosome secretion assay for HCT8 cells transfected with the above two vectors. All results are representative of three independent experiments, and shown as mean ± SD (**A**, **D**, **F**, **G**, **I**).

The clinical data showed that circSKA3 was upregulated in CRC tissues but downregulated in serum exosomes of CRC patients. Moreover, circSKA3 is known to play an oncogenic role in CRC. Our previous study has revealed that CRC cells actively excrete tumor-suppressive circRNAs in exosomes to sustain cancer cell fitness [5]. However, it remains unknown whether CRC cells preferentially retain oncogenic circRNAs to maintain their malignant behavior. To find out, we first used a nucleoplasm separation kit to confirm that circSKA3 is located mainly in the cytoplasm (Fig. 4A). Next, we isolated exosomes via ultracentrifugation in different cell lines and identified them by cryo-electron microscopy (Fig. 4B) and immunoblotting to detect the exosome-specific markers CD63 and TSG101 (Fig. 4C). Then, we found that overexpressed circSKA3 was still preferentially retained in the cytoplasm in the CRC cell lines HCT116, SW480 and HCT8, while in normal cell lines (NCM460 and 293T), it was excreted within exosomes (Fig. 4D).

Recently, the specific cellmotifs or exomotifs in miRNAs that determine intracellular retention or exosomal secretion were identified in different types of tissues [14]. Therefore, we wanted to determine whether there were specific motifs in circSKA3 that regulate its retention in CRC cells. Thus, we produced four truncated circSKA3 expression vectors with 100 nt deletion segments (Fig. 4E). When these vectors were transfected into HCT116 cells, the levels of circSKA3 with deletion of nucleotides 276–378 nt (Δ4) were lower in the cytoplasm but greater in exosomes than the levels of the FL and other truncated circSKA3 (Δ1, Δ2, and Δ3) (Fig. 4F). To further identify the cellmotif in circSKA3, we constructed another two circSKA3 expression vectors with deletion of nucleotides 276–312 (Δ4-1) and nucleotides 313–378 (Δ4-2). Compared with the FL sequence and the Δ4-1 vector, the vector with the deletion of nucleotides 313–378 nt (Δ4-2) resulted in significantly reduced intracellular retention of circSKA3 (Fig. 4G). Fortunately, we found a cellmotif (AGAAC) that has been reported to regulate the intracellular retention of miRNAs in the segment of circSKA3 from nucleotides 313–378 (Fig. 4H). Deleting this cellmotif decreased the intracellular retention of circSKA3 and increased the exosomal secretion of circSKA3 (Fig. 4I), consistent with previous findings regarding miRNA intracellular retention and exosome secretion. Together with the previous data, our findings suggest that oncogenic circSKA3 can be retained in CRC cells and that a specific cellmotif can facilitate the intracellular retention of oncogenic circRNAs.

### CircSKA3 promotes epithelial–mesenchymal transition (EMT) by interacting with SLUG in CRC

**Fig. 5:**
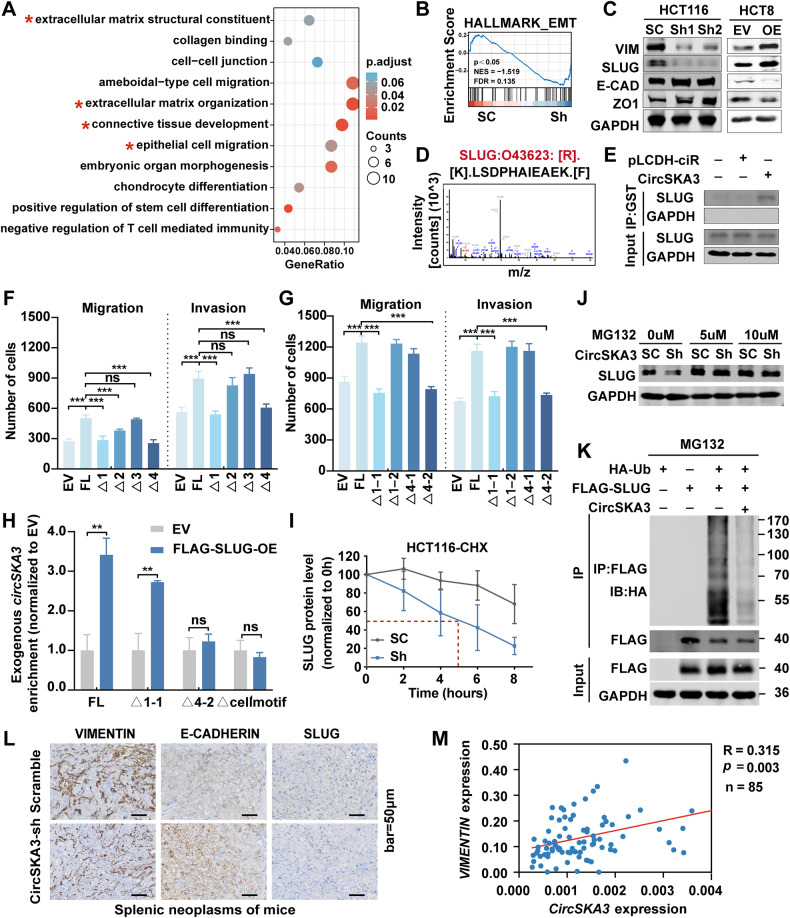
CircSKA3 promotes EMT in CRC by stabilizing SLUG. **A** Top biological processes regulated by circSKA3 as determined by GO enrichment analysis of the differentially expressed genes (DEGs) based on the Database for Annotation, Visualization and Integrated Discovery (DAVID) online tools (|log2(fold-change)| >1 and *p* ≤ 0.05). **B** GSEA of circSKA3-knockdown HCT116 cells. **C** Detection of EMT-related markers in circSKA3-knockdown HCT116 cells and circSKA3-overexpressing HCT8 cells by immunoblotting. **D** Amino acid sequence of SLUG as detected by MS. **E** Detection of SLUG in RNA pulldown assays by immunoblotting. **F**, **G** Quantitative analysis of the results of a transwell assay for the migration and invasion abilities of HCT8 cells after overexpression of truncated circSKA3 and FL circSKA3. **H** Detection of the enrichment of FL, Δ1-1, Δ4-2, and Δcellmotif-truncated circSKA3 in the RIP assay by RT‒qPCR. **I** Statistical chart of SLUG protein levels at different time points after CHX treatment of control and knockdown HCT116 cells. **J** Detection of SLUG protein levels in circSKA3-knockdown and control HCT116 cells treated with different concentrations of MG132 by immunoblotting. **K** Detection of the ubiquitination level of SLUG by IP assay. **L** Detection of E-CADHERIN, VIMENTIN, and SLUG expression in a mouse model of CRC spleen-liver metastasis (scale bar=50 μm). **M** Correlation between circSKA3 and VIMENTIN mRNA levels in human CRC samples. All results are representative of three independent experiments, and shown as mean ± SD (**H**) or mean ± SEM (**F**, **G**, **I**). Statistical significance was accessed by Student’s *t*-test (**H**), one-way ANOVA (**F**, **G**), and Pearson’s correlation analysis (**M**). ns, not significant with *p* > 0.05, *p < 0.05, **p < 0.01, ***p < 0.001.

To clarify the specific mechanism by which circSKA3 promotes the progression of CRC, we performed mRNA sequencing on stable circSKA3-knockdown HCT116 cells. The results showed that circSKA3 knockdown caused 143 genes to be downregulated and 100 genes to be upregulated (Fig. S4A). GO enrichment revealed that these differentially expressed genes were enriched with EMT-related pathway terms such as the extracellular matrix structural constituent, extracellular matrix organization and connective tissue development terms (Fig. 5A). GSEA revealed that circSKA3 knockdown was also significantly associated with EMT pathways (Fig. 5B). Immunoblotting showed that circSKA3 knockdown decreased the expression of mesenchyme-related markers, such as VIMENTIN and SLUG, while increasing the expression of epithelium-related markers, such as E-CADHRIN and ZO1; however, the opposite results were observed in circSKA3-overexpressing HCT8 cell lines (Fig. 5C). These data further demonstrate that circSKA3 promotes EMT in CRC cells.

Recently, some circRNAs have been reported to function by encoding short peptides [15]. To verify whether circSKA3 promotes EMT in CRC through similar mechanisms, we analyzed the circSKA3 sequence using the TransCirc website, which showed an overlapping open reading frame (ORF) and internal ribosomal entry site (IRES) in circSKA3 (Fig. S4B). Therefore, we cloned the FLAG sequence into the circSKA3 overexpression vector (Fig. S4C). After these vectors were transfected into 293 T cells, no protein bands were detected by immunoblotting (Fig. S4D), demonstrating that circSKA3 does not play a role in CRC by encoding a protein.

To further clarify the molecular mechanisms of circSKA3 in CRC progression, we co-transfected MS2-labeled circSKA3 with the fusion MS2-GST into 293 T cells (Fig. S4E). Then, GST pulldown assays were performed, and circSKA3 was confirmed to be enriched by RT‒qPCR and immunoblotting (Fig. S4F and S4G). Fortunately, we identified SLUG as a binding protein of circSKA3 by MS (Figs. S4H and 5D), and the interaction was further verified by RIP assay (Fig. 5E).

Furthermore, we transfected the above truncated circSKA3 vectors (Fig. 4E) into HCT8 cells to identify the motif in circSKA3 that functions in tumor promotion by binding to SLUG. As shown in Fig. 5F and S4I, FL circSKA3 promoted CRC cell migration and invasion. The constructs with deletion of nucleotides 379–412/1–80 (Δ1) and nucleotides 276–378 (Δ4) significantly reversed the changes in the migration and invasion abilities, while the construct with deletion of nucleotides 81–178 nt (Δ2) only slightly reduced migration and invasion abilities, and the construct with deletion of nucleotides 179–275 (Δ3) had no effects. Furthermore, compared with the FL, 81–178 (Δ1-2) and 276–312 (Δ4-1) sequence, the constructs with deletion of nucleotides 379–412/1–23 (Δ1-1) and 313–378 (Δ4-2) significantly inhibited migration and invasion (Figs. 5G and S4J). Collectively, the data indicate that the 1-1 and 4-2 motifs might be the key regulatory domains of circSKA3 that play roles in promoting CRC metastasis.

To investigate whether the SLUG interaction site is located in the 1-1 or 4-2 motifs of circSKA3, we performed exogenous RIP assays in 293 T cells overexpressing the FL, Δ1-1, Δ4-2, and Δcellmotif (Fig. 4I) vectors. In addition to FL circSKA3, Δ1-1 circSKA3 could still bind to SLUG, but deletion of the 4-2 motif abolished the interaction of SLUG with circSKA3; more intriguingly, deletion of the cellmotif element (Δcellmotif) in the 4-2 motif also blocked SLUG from binding to circSKA3 (Figs. 5H and S4K). Therefore, the cellmotif element mediated not only intracellular circSKA3 retention but also the circSKA3–SLUG interaction. Nevertheless, it remained unknown how circSKA3 regulates SLUG in CRC progression. Since circSKA3 could bind to the SLUG protein while knockdown of circSKA3 inhibits SLUG expression at the protein level, we speculated that the interaction of circSKA3 with SLUG might stabilize the protein level of SLUG. Therefore, we used cycloheximide (CHX) tracking assays to analyze the stability of SLUG, and the results showed that the half-life of SLUG was significantly reduced in circSKA3-knockdown CRC cells (Figs. 5I and S4L). The proteasome inhibitor MG132 reversed the decrease in SLUG protein level caused by circSKA3 knockdown (Fig. 5J). To test whether circSKA3 promoted SLUG protein stability by regulating ubiquitination, we co-transfected HA-Ub, FLAG-SLUG, and circSKA3 expression vectors into 293 T cells and then treated the cells with MG132 for 6 h. An IP assay showed that the ubiquitination degradation of SLUG was significantly inhibited by circSKA3 overexpression (Fig. 5K). These data indicate that circSKA3 can stabilize SLUG by blocking ubiquitination.

Next, we detected the expression of VIMENTIN, E-CADHERIN, and SLUG in orthotopic lesion sections of mouse CRC spleen-liver metastasis models by IHC, which showed that E-CADHERIN levels were increased and that VIMENTIN and SLUG levels were significantly decreased in the circSKA3-knockdown group (Fig. 5L). Finally, the VIMENTIN level was analyzed in 85 CRC samples by RT‒qPCR. The results revealed that circSKA3 levels were positively correlated with VIMENTIN expression (*p* = 0.003) (Fig. 5M). Together with the above data, circSKA3 promotes EMT and metastasis in CRC by inhibiting SLUG ubiquitination degradation.

### FUS regulates the circularization of circSKA3 by binding to a specific motif

**Fig. 6:**
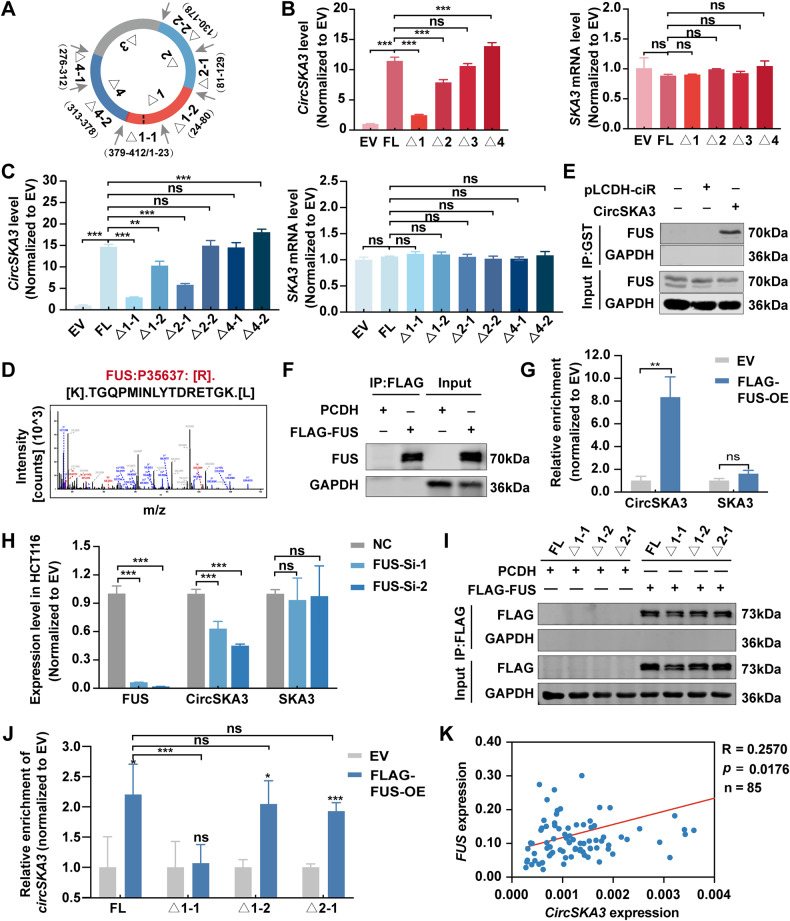
FUS regulates the circularization of circSKA3 via a specific motif. **A** Schematic diagram of truncated circSKA3 plasmid construction. **B**, **C** Circularization efficiency of circSKA3 and the linear SKA3 level. **D** Amino acid sequence of FUS identified by MS. **E** Detection of FUS in the GST-RNA pulldown assay by immunoblotting. **F** Detection of SLUG in the RIP assay by immunoblotting. **G** Detection of circSKA3 enrichment in the RIP assay by RT‒qPCR. **H** Detection of the mRNA expression of FUS, circSKA3, and SKA3 by RT‒qPCR. **I** Detection of FLAG in the RIP assay by immunoblotting. **J** Detection of truncated circSKA3 enrichment in the RIP assay by RT‒qPCR. **K** Correlation between circSKA3 and FUS mRNA levels in human CRC samples. All results are representative of three independent experiments, and shown as mean ± SD (**B**, **C**, **G**, **H**, **J**, **K**). Statistical significance was accessed by Student’s *t*-test (**G**), one-way ANOVA (**B**, **C**, **H**, **J**), and Pearson’s correlation analysis (**K**). ns, not significant with *p* > 0.05, *p < 0.05, **p < 0.01, ***p < 0.001.

CircRNA back-splicing is regulated not only by the complementary elements of the flanking introns but also by the self-motifs in the exons [16]. To identify the self-motifs that regulate circSKA3 circularization, we transfected the above truncation vector of circSKA3 into CRC cells and then examined the efficiency of circSKA3 circularization (Fig. 6A). We found that the circularization efficiency of circSKA3 was significantly decreased after deletion of nucleotides 379–412/1–80 (Δ1) and nucleotides 81–178 (Δ2) of circSKA3, while the circularization efficiency of circSKA3 was increased after deletion of nucleotides 276–378 (Δ4) segments (Fig. 6B). In addition, it was verified that none of these truncations affected the expression of the host gene SKA3 (Fig. 6B).

Furthermore, the circularization efficiency of circSKA3 was significantly decreased after nucleotides 379–412/1–23 (Δ1-1) were deleted, and the deletion of nucleotides 24–80 (Δ1-2) and nucleotides 81–129 (Δ2-1) partially inhibited circularization of circSKA3. However, circularization was increased by the deletion of nucleotides 313–378 (Δ4-2). None of the truncation vectors changed SKA3 mRNA levels (Fig. 6C). These results suggest that the 1-1, 1-2, and 2-1 segments are cis-elements that positively regulate circSKA3 circularization in its exon, while the 4-2 segment is the negative regulatory element.

To further illustrate the circularization mechanism of circSKA3, we predicted RBPs that bind to circSKA3 with the circAtlas website (http://circatlas.biols.ac.cn/). Combined with the above MS data, the results indicated that the multifunctional DNA/RNA-binding protein FUS is an RBP of circSKA3 (Fig. 6D). Then, a GST-RNA pulldown assay confirmed that circSKA3 interacts with FUS (Fig. 6E). Reciprocally, a RIP assay showed that circSKA3, but not SKA3, was significantly enriched by anti-FLAG beads in HCT116 cells with FLAG-labeled FUS overexpression (Fig. 6F, G). Moreover, FUS knockdown inhibited the expression of circSKA3 but not SKA3 (Fig. 6H). Interestingly, RIP assays showed that FUS could bind to FL, Δ1-2, and Δ2-1 circSKA3 but not to Δ1-1 circSKA3, which indicates that FUS positively regulates the circularization of circSKA3 by interacting with the 1-1 motif (Fig. 6I, J). Furthermore, we detected the mRNA levels of circSKA3 and FUS in 85 CRC samples and observed a significant positive correlation between circSKA3 and FUS (Fig. 6K). Taken together, these data demonstrate that FUS promotes the circularization of circSKA3 by binding to the 1-1 motif (nucleotides 379–412/1–23) of circSKA3 as a positive regulatory element.

### ASOs targeting the circularization element and cellmotif inhibit CRC metastasis

**Fig. 7:**
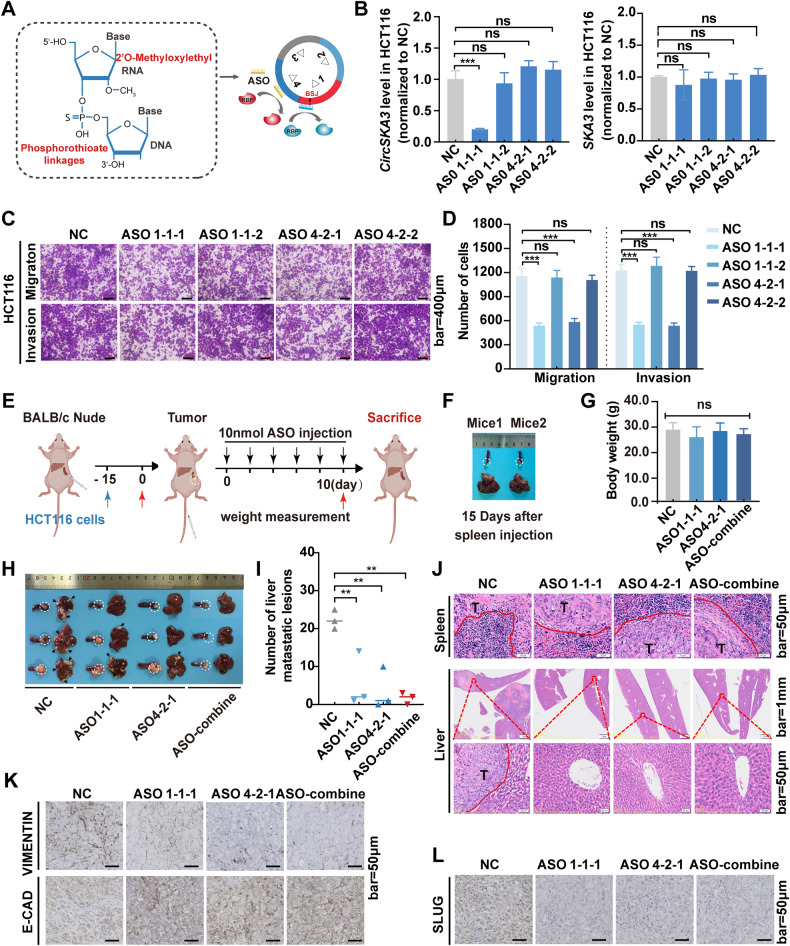
The ASOs inhibit CRC metastasis in vitro and in vivo by regulating circSKA3 circularization and blocking the functional motif. **A** Schematic diagram of the functional mechanism and chemical modification of ASOs. **B** Expression levels of circSKA3 and SKA3 in the ASO-NC group and the groups treated with ASOs targeting specific circSKA3 motifs in HCT116 cells. **C**, **D** Transwell assay and quantitative analysis of the migration and invasion abilities of ASO-treated HCT116 cells (scale bar = 400 μm). **E** Schematic diagram of ASO treatment in the spleen-liver metastasis model via tail vein injection (*n* = 3). Blue arrows represent the time point of tumor modeling, black represents the time point of administration of ASO and the time point of detection of mouse body weight, and red represents the time point of sacrificing mice (Created with BioRender.com). **F** Images of the spleen and liver after in situ injection of HCT116 cells for 15 days. **G** Body weights of nude mice after ASO treatment. **H** Images of the spleens and livers of nude mice treated with ASOs six times. **I** Number of distant liver metastatic lesions. **J** H&E staining of splenic neoplasms and liver metastatic foci. **K**, **L** E-CADHERIN and VIMENTIN and SLUG expression in xenograft tumors after ASO treatment (scale bar = 50 μm). All results are representative of three independent experiments, and shown as mean ± SD (**B**, **G**, **I**) or mean ± SEM (D). Statistical significance was accessed by one-way ANOVA (**B**, **D**, **G**, **I**). ns, not significant with *p* > 0.05, **p < 0.01, ***p < 0.001.

Our previous results clarified that the splicing factor FUS promotes circSKA3 circularization by binding to a specific regulatory element, so we hypothesized that blocking the interaction between FUS and circSKA3 might inhibit CRC metastasis. Similarly, we hypothesized that abolishing the binding of SLUG to the cellmotif in circSKA3 might be an alternative therapy for CRC metastasis. To test this hypothesis, we designed and synthesized ASOs specifically targeting the circularization regulatory element (ASO1-1-1 and ASO1-1-2) and cellmotif (ASO4-2-1 and ASO4-2-2) with full-chain phosphorothioate and five 2’-O-Me-substituted oligonucleotide fragments at both ends (Fig. 7A). When these ASOs were transfected into HCT116 cells, ASO1-1-1 significantly inhibited the expression of circSKA3, while the other ASOs did not obviously change the expression of circSKA3; none of the ASOs affected SKA3 mRNA expression (Fig. 7B). Next, transwell assays were performed, which showed that not only ASO1-1-1 but also ASO4-2-1 could significantly suppress CRC cell migration and invasion (Fig. 7C, D). These findings suggested that ASO1-1-1 inhibited circSKA3 circularization and that ASO4-2-1 blocked circSKA3 from interacting with SLUG.

To explore the therapeutic effects of the ASOs in vivo, we constructed a mouse liver metastasis model through spleen injection of HCT116 cells (Fig. 7E). Preliminary experiments suggested that orthotopic tumors formed in the mice without distant metastasis on the 15^th^ day after CRC cell injection (Fig. 7F). After 15 days, we injected ASOs via the tail vein once every two days for six cycles. However, there was no significant difference between the ASO therapy group and the control (NC) group in mouse body weight on the 45th day (Fig. 7G), which might have been explained by the cachexia in the tumor-bearing mice. As expected, the numbers of liver metastases in the 10 nmol ASO1-1-1 and ASO4-2-1 treatment groups were significantly reduced. Surprisingly, there were no obvious liver metastases in the combined-ASO group (Fig. 7H, I). H&E staining showed that the mice treated with ASOs presented less invasive edges in their spleen orthotopic tumors and smaller metastatic foci in their livers (Fig. 7J). IHC revealed that each ASO inhibited VIMENTIN and SLUG expression and enhanced E-CADHERIN expression and that combination treatment with both ASOs also exerted significant effects on E-CADHERIN, VIMENTIN and SLUG (Fig. 7K, L). These in vivo data further support the idea that the specific ASOs can inhibit CRC EMT and metastasis by repressing circSKA3 circularization or the circSKA3–SLUG interaction.

## Discussion

CRC still ranks third among tumors in terms of morbidity and mortality, and metastasis is the major cause of death. Our previous study revealed similar mutation profiles between CRC primary foci and metastatic foci, consistent with the findings of research by other groups [17, 18]. Therefore, CRC metastasis might be regulated mainly by epigenetics rather than genetics, because metastasis-private mutations are not drivers of cancer spread [18]. CircRNAs are important epigenetic regulators. Recently, some metastasis-associated circRNAs have been reported, for example, high levels of circErbin and circLONP2 in CRC promote tumor growth and metastasis [19, 20], while tumor-suppressive circRNAs such as circPPFIA1, circ_0009361, and circ_0021977 can enhance tumor-suppressor gene expression to inhibit CRC metastasis [21, 22]. Here, we identified circSKA3 as a prometastatic circRNA that was upregulated in CRC tumor tissues and promoted CRC metastasis. EMT has been recognized as a classic mechanism of tumor metastasis that can be regulated by various circRNAs [23, 24]. For example, CiRS-7 and circCER regulate EMT via ceRNA [25, 26], circUSUN2 promotes CRC liver metastasis by stabilizing high-mobility group A2 [27], and circSPARC regulates the JAK2-STAT3 signaling pathway by binding to miR-485-3p and FUS to promote CRC metastasis [28]. Our in vitro and in vivo data confirmed that circSKA3 could also promote EMT to accelerate CRC metastasis by binding to SLUG. Together with SNAI1, SLUG (also named SNAI2) belongs to the snail family, which contains the most well-studied EMT core transcription factors. The members of this family are regulated by various signals, such as TGF-β, notch, wnt, reactive oxygen species, and hypoxic stress [29]. However, SLUG, with its short half-life, is exquisitely regulated by proteasomal degradation mediated by the E3 ubiquitin ligases FBXL14 and FBXW1 [30]. In the present study, the functional motif of circSKA3 was located at nucleotides 313–378 (4-2) and found to mediate the interaction of circSKA3 with SLUG to inhibit the ubiquitination and degradation of SLUG. These data suggest that circSKA3 accelerates CRC EMT and metastasis by enhancing SLUG protein stability.

Similar to many linear mRNAs, circRNAs with intron retention are preferentially sequestered in the nucleus; however, many circRNAs resulting from exon back-splicing are frequently located in the cytoplasm [16]. Consistent with this, circSKA3 derived from exon 4 of SKA3 was mainly distributed in the cytoplasm and was highly expressed in CRC tissues. However, the level of circSKA3 was unexpectedly decreased in serum from CRC patients. We hypothesized that this contradictory expression pattern was attributable to the preferential retention of oncogenic circSKA3 in the cytoplasm of tumor cells to maintain their fitness, which complemented our previous hypothesis that tumor cells tend to expel tumor-suppressive circRNAs via exosome secretion [5]. The specific motifs of circSKA3 for regulating cytoplasm retention were identified at nucleotides 313–378. Interestingly, the cellmotif (AGAAC) at nucleotides 313–378 was verified as the key element for the retention of circSKA3 inside CRC cells; this motif has been previously reported to mediate intracellular miRNA retention [14]. More interestingly, this cellmotif is the functional sequence by which circSKA3 binds to SLUG to play a role in promoting cancer. Therefore, we reasonably infer that SLUG binds to the key cellmotif of circSKA3 to prevent it from being sorted into exosomes and secreted from CRC cells. Our findings suggest that tumor cells “intelligently” retain the beneficial circSKA3 in their cytoplasm to sustain their migration and invasion characteristics.

Currently, circRNAs are thought to be generated by alternative back-splicing of pre-mRNAs [31], which is mainly regulated by flanking intron complementary elements and trans-acting factors (RBPs), such as FUS [32], adenosine deaminase acting on RNA1 [33] and DEAH-box helicase 9 [34]. Here, we showed that truncation of nucleotides 379–412/1–23 (Δ1-1) of circSKA3 significantly reduced circSKA3 circularization efficiency without affecting the transcription level of the host gene SKA3 and that deletion of nucleotides 81–129 (Δ2-1) also reduced the circularization efficiency of circSKA3 by approximately 50%. However, the BSJ site of circSKA3 is located in the segment encompassing nucleotides 379–412/1–23. Interestingly, FUS, an important circRNA circularization regulator, can bind to nucleotides 379–412/1–23 to promote circSKA3 circularization, which has been reported to be involved in the regulation of the circularization of multiple circRNAs, such as circEZH2 [35], circPDE4B [36] and circRHOBTB3 [37]. Based on the sequence of circSKA3 from nucleotides 81 to 129, there might be an alternative exon splicing enhancer that regulates circSKA3 circularization [38], which needs to be confirmed in the future. As the deletion of nucleotides 313–378 (Δ4-2) increased circularization efficiency, we infer that the deletion of this segment caused cellmotif deficiency to decrease circSKA3 retention in cells. However, the possibility that a negative regulatory element exists in the 313–378 sequence cannot be ruled out. These data suggest that there are circularization elements in circSKA3 exons that regulate the expression of circSKA3 in CRC cells.

The earliest ASO drug on the market, Vitravene, was approved by the FDA in 1998 for the treatment of cytomegalovirus retinitis complicated by AIDS [39]. In addition to ASOs for hereditary diseases such as Duchenne muscular dystrophy and spinal muscular atrophy, a number of ASO drugs for the treatment of hepatitis B, fatty liver disease and cardiovascular diseases are also in the clinical research stage [40]. With improvements in chemical modification methods and the development of drug delivery systems, the application of ASOs is expanding. ASOs can directly bind to RNA, not only inhibiting target genes but also upregulating their expression. ASOs are small fragments of single-stranded RNA with reduced hydrophilicity that can diffuse well in cells and tissues. Because ASOs work by directly binding to mRNA, they have short half-lives and few side effects. Here, we designed second-generation ASOs modified by 2′-O-methyl (2′-OME) phosphorothioate with a length of 20 nt that targeted the functional motif mediating the circSKA3–SLUG interaction and the circularization elements that bind to FUS to regulate circSKA3 expression. Our results showed that these ASOs not only inhibited the migration and invasion potential of CRC cells in vitro but also significantly decreased the number and size of liver metastatic foci in vivo. As expected, the combined use of the two kinds of ASOs significantly enhanced the tumor-suppressive effect. Under the premise of a comprehensive understanding of the oncogenic mechanism of circSKA3 in CRC progression, we have reason to believe that ASOs targeting different functional motifs of circSKA3 could become treatment options for CRC.

**Fig. 8:**
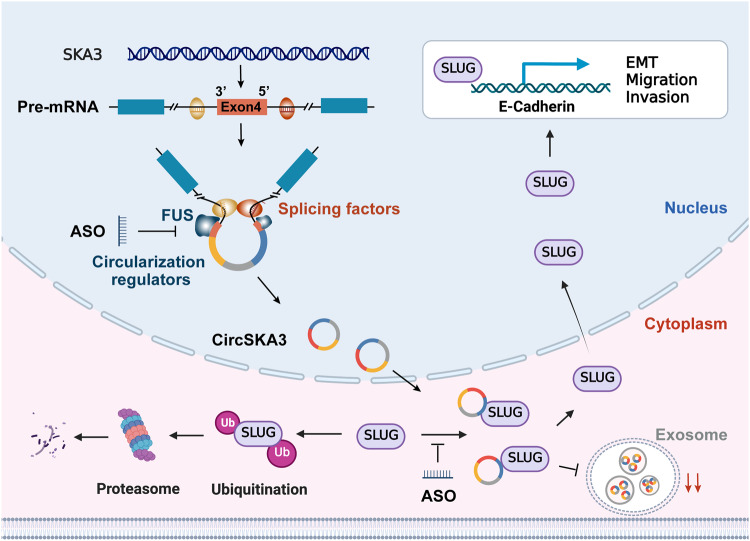
CircSKA3 promotes CRC progression by attenuating the ubiquitination and degradation of SLUG.

In conclusion, CRC cells might retain circSKA3 in the cytoplasm through a specific cellmotif to inhibit exosomal secretion, and intracellular circSKA3 can block SLUG ubiquitination degradation and promote CRC metastasis. Furthermore, specific ASOs targeting the key circularization element and functional motif of circSKA3 could become promising therapeutic agents for CRC (Fig. 8).

## Materials and methods

### Clinical samples

Colorectal adenocarcinoma and normal tissue paired samples (*n* = 55), as well as another 27 tumor tissue samples from colorectal adenocarcinoma patients, were obtained from patients during surgery at Run Run Shaw Hospital Affiliated to Zhejiang University School of Medicine. Serum samples from 105 normal donors and 72 CRC patients were also obtained from physical examinations and from in patients at Run Run Shaw Hospital Affiliated with Zhejiang University School of Medicine. Ten paired normal–adenoma/adenocarcinoma samples for RNA-seq were obtained from the First Affiliated Hospital of Zhejiang University School of Medicine (Ethics Committee number: 2021–021). All patients were informed, and none were involved in the design, conduct, reporting, or dissemination plans of our research.

### Public dataset analysis

The total RNA expression data of serum exosomes from 32 healthy donors and 12 CRC patients were obtained from the GSE100206 and GSE100063 datasets in the Gene Expression Omnibus (GEO) database. We calculated the AUC using serum samples from 32 healthy donors and 12 CRC patients. All these datasets were analyzed on the GPL11154 platform (Illumina HiSeq 2000). A *p* < 0.05 and a fold-change > 1.2 were considered to indicate statistical significance.

### Cell lines and cell culture

The human CRC cell lines HCT116, SW620, HCT8, and SW480 and the human normal liver cell line L02 were purchased from the American Type Culture Collection (ATCC, Manassas, VA, USA). 293T, human embryonic lung fibroblasts (Helf), and cancer-associated fibroblasts (Caf) cells were purchased from the National Biomedical Experimental Cell Resource Bank (Shanghai, China). The normal colorectal epithelial cells (NCM460) were purchased from EK Bioscience (Shanghai, China). The 293T and L02 cells were cultured in DMEM (Gibco). The HCT116, SW620, HCT8, SW480 and NCM460 cells were maintained in RPMI 1640 medium (Gibco). Ten percent fetal bovine serum (Gibco) and 1% penicillin‒streptomycin was added to all media. All cells were incubated at 37 °C under 5% CO_2_ in an incubator. Exosome-free FBS was obtained by ultracentrifugation according to Beckman’s official procedure (https://www.mybeckman.cn/support/technical-documents).

### RNA isolation and RT‒qPCR

Total RNA from cells or mouse tissues was isolated using TRIzol Reagent (Invitrogen, CAT#15596018). The exosomal and serum total RNA of humans and mice was isolated using TRIzol LS (Invitrogen, CAT#10296010) and measured with a NanoDrop (Thermo Scientific). cDNA was synthesized by HiScript II Reverse Transcriptase (Vazyme, CAT#R223-01) and quantified by qPCR using SYBR qPCR Master Mix (Vazyme, CAT#Q711-02).

### RNase R digestion

After extracting the total RNA of HCT116 and SW620 cells and determining the concentration, 1.5 μg of RNA was added to 1.5 U of RNase R, digested at 37 °C for 30 min, and then digested at 70 °C for 10 min. The reverse transcription product was electrophoresed with a 1% TBE gel to analyze the target band changes and quantitatively analyzed by RT‒qPCR.

### Actinomycin D assay

HCT116 and SW620 cells were cultured with 2 μg/ml actinomycin D (MCE, CAT#50-76-0), and the cells were harvested at different time points. After RNA extraction, RT‒qPCR was performed to examine circRNA levels. GAPDH was used as an internal reference, and the data at each time point were normalized to those at 0 h.

### nuclear and cytoplasmic RNA isolation

For nuclear and cytoplasmic RNA isolation, a PARIS kit was used according to the manufacturer’s instructions (Ambion CAT#AM1921) [41]. 1 × 10^7^ HCT116 and HCT8 cells were washed with PBS, resuspended them in 400 μl of cell fraction buffer, and placed them on ice for 10 min to lyse the cell membrane. After centrifugation, the supernatant containing the cell lysate was collected. Next, we resuspended the pellet in 400 μl of cell fraction buffer, vigorously shaken, and then incubated it on ice for 10 min to lyse the cell nucleus. Finally, RNA was extracted from the obtained lysates following the instructions of the kit and measured the RNA level using RT-qPCR. GAPDH and β-ACTIN were used as cytoplasmic references, while NEAT1 and MALAT1 were used as nuclear references, to calculate the expression levels of the target gene in different compartments [5].

### Transfection of vectors and ASOs

All siRNA, shRNA, gRNA, ASO, and primer sequences are shown in Table S1. The vectors are shown in Table S2. The siRNAs and ASOs used in our research were synthesized by GenePharma (Shanghai, China). The ASOs with a full-chain phosphorothioate and five 2’-O-Me-substituted oligonucleotide fragments at both ends used in vivo in mice were synthesized by RiboBio company (Guangzhou, China). Plasmids were transfected into cells using Lipofectamine 2000 (Invitrogen, CAT#11668019), LipD293 (SignaGen, CAT#SL100668), and Neofect (Neofect, CAT#TF20121201). siRNAs and ASOs were transfected into cells using GenMute siRNA transfection reagent (SignaGen, CAT#SL100568). All assays were performed after 48 h of transfection.

### Cell proliferation, migration, and invasion assays

Cell proliferation experiments were performed using a Cell Counting Kit-8 (CCK-8) (Boster, CAT#AR1160). A total of 2000 cells were seeded into each well of a 96-well plate and examined at the same time every day. Ten microliters of CCK-8 reagent were added during detection. After 2 h of incubation at 37 °C, 80 μl was aspirated, and the absorbance at 450 nm was measured. Five replicate wells were made for each group, and each experiment was repeated 3 times. For migration experiments, 1×10^5^ HCT116, HCT8, and SW480 cells and 2 × 10^5^ SW620 cells were plated into the upper chamber of an 8 μm transwell chamber (Corning Costar, CAT#3422). For invasion experiments, 50 μl of diluted Matrigel (Corning, BD Matrigel, CAT#356234) was added before plating cells in a transwell chamber. Then, 600 μl of medium containing 10% FBS was added to the lower chamber. After 24–48 h of culture, the chambers were fixed with 4% paraformaldehyde and stained with crystal violet. After the chambers were air-dried, photographs were taken, and the percentages of positive cell area were quantified using ImageJ software (1.49 v). Three fields were observed per chamber, and the experiment was repeated three times.

### Immunoblotting

RIPA buffer containing the protease inhibitor PMSF was added to the cell pellets, which were lysed by noncontact sonication. The protein concentration was determined using a BCA quantification kit (Thermo Scientific, CAT#23225). The protein bands were separated by SDS‒PAGE and transferred to nitrocellulose membranes (Millipore, USA). After blocking with 5% nonfat milk in TBST for 1 h, the membranes were incubated with primary antibodies against SKA3 (Thermo, CAT#A304-214A-T, 1:1000), Vimentin (CST, CAT#D21H3, 1:1000), E-Cadherin (CST, CAT#14472S, 1:1000), Slug (CST, CAT#9585, 1:1000), Zo1 (CST, CAT#13663 S, 1:1000), Fused in sarcoma (FUS) (Proteintech, CAT#KHC0057, 1:1000), Flag (Sigma, CAT#F2555, 1:1000), and GAPDH (CST, CAT#5174 S, 1:1000) overnight at 4 °C. After incubation with the fluorescent secondary antibodies for 1 h, the target bands were detected by an Odyssey instrument (LI-COR, USA).

### Animal experiments

All animal experiments were in accordance with the protocol approved by the Institutional Animal Care and Use Committee of Zhejiang University (Ethics Committee number: 12169), and all operations were carried out in accordance with the operating standards of the Zhejiang University Experimental Animal Center. Healthy male BALB/c nude mice (age: 5 weeks, weight: 16–18 g) were purchased from Jiangsu Gempharmatech Co. Ltd. and fed under SPF conditions at the Laboratory Animal Center, Zhejiang University.

#### Liver metastasis models

Fourteen mice were randomly divided into two groups. A total of 1 × 10^6^ circSKA3-scramble and circSKA3-shHCT116 cells were resuspended in 100 μl of PBS with 10% Matrigel and injected into the spleens of nude mice in situ. On the 45th day of modeling, the mice were sacrificed, and their spleens and livers were collected. Then, the liver metastases were counted and measured before images were obtained. Finally, the cells were fixed with 4% paraformaldehyde for pathological staining.

#### ASO-injected models

A total of 1 × 10^6^ HCT116 cells were resuspended in 100 μl of PBS supplemented with 10% Matrigel and injected into the spleens of 14 nude mice. On the 15th day of modeling, two randomly selected mice were sacrificed to confirm that the spleens had formed tumors in situ, but no liver metastases were found. 12 mice had spleen tumors were randomly divided into 4 groups. A total of 6 doses of 10 nmol of control, 10 nmol of ASO1-1-1, 10 nmol of ASO4-2-1, and 20 nmol of ASO1-1-1 + ASO4-2-1 combined were administered by tail vein injection once every two days. On the 45th day of modeling, the mice were sacrificed, and their spleens, livers, and blood were collected, photographed, and stored for future use.

### Histological analysis

Mouse liver and spleen samples were fixed with 4% paraformaldehyde and cut into 4 μm thick sections. hematoxylin and eosin (H&E) staining and immunohistochemistry (IHC) were performed as previously described [42]. The primary antibodies used were against vimentin (CST, CAT#D21H3, 1:400), E-cadherin (CST, CAT#14472S, 1:400), and Slug (CST, CAT#9585, 1:200).

### Isolation and identification of cellular exosomes

CRC cells were plated in a 15 cm cell culture dish, 10% exosome-free FBS was added to the medium, and the cells were cultured at 37 °C for 48 h. The cells were grown to 80% confluence, and the cell supernatant was collected. The cell supernatant was centrifuged at 1500 rpm for 5 min to isolate viable cells. The supernatant was then collected and centrifuged at 1500 × *g* for 10 min to remove cellular debris. The resulting supernatant was collected and centrifuged at 14,000 × *g* for 60 min to remove microvesicles. After the supernatant was collected and passed through a 0.22 μm filter, the exosomes were collected by ultracentrifugation at 25,300 rpm for 2 h. The supernatant was discarded, and 200 μl of PBS was added to resuspend the pellet. After adding 4x SDS loading buffer-boiled protein, immunoblotting was used to identify the exosome-related markers TSG101 (Proteintech, CAT#28283-1-AP, 1:400) and CD63 (CST, CAT#52090S, 1:500). A Tecnai G2 Spirit 120 kV transmission electron microscope (FEI, Hillsboro, USA) was used to identify exosomes.

### Exosomal secretion assay

The full-length (FL) circSKA3 and constructed circSKA3 overexpression vectors were transfected into HCT8 cells with a confluence of 40% in a 10 cm dish for 48 h, after which the cells were transferred to a 15 cm dish. After another 48 h of culture, the cells and culture supernatant were collected. The cells were directly treated with TRIzol to extract intracellular RNA, the supernatant was collected to obtain exosomes according to the above steps, and the RNA in the exosomes was extracted with TRIzol LS. Primers were designed on the CopGFP sequence of pLCDH-ciR as an internal control to calculate the exosome secretion efficiency with the following formula:

$${\rm{Exosome\; secretion\; efficiency}}=\frac{{\rm{Exosome}}({\rm{Exp}}{\rm{OE}}/{\rm{Exp}}{\rm{EV}})}{{\rm{Cytosome}}({\rm{Exp}}{\rm{OE}}/{\rm{Exp}}{\rm{EV}})}$$

### RNA-seq and Gene set enrichment analysis (GSEA)

Total RNA was isolated from tissues using Magzol Reagent (Magen, China) according to the manufacturer’s protocol. CIRI2 and CIRCexplorer2 were used to detect circRNAs. The reads were mapped to the human reference genome GRCh37/hg19 (http://genome.ucsc.edu/) by BWA-MEM or TopHat. CIRI2 detects the paired chiastic clipping signals from the mapping information of reads. CIRCexplorer2 uses TopHat and TopHat-Fusion alignment output to detect circRNAs. If a circRNA was detected by both methods, it was considered a candidate circRNA. The DEseq/DESeq2/edgeR/DEGseq packages were used to determine differentially expressed circRNAs according to the criteria of a |log2(FC)| >1 and *p* ≤ 0.05.

RNA was extracted from HCT116-SC and HCT116-sh cells, and the sequencing and preliminary data analysis were performed with assistance by Bioacme (Wuhan, China). Each set of samples had 3 biological replicates. Sequencing and data analysis were performed as previously described [5]. An adjusted p value of a gene<0.05 and a log2(FC) > 1 were used as the criteria for differentially expressed genes (DEGs) for further data analysis. A gene expression heatmap was generated with an R package (v3.6.3). Gene Ontology (GO) enrichment was performed using the Database for Annotation, Visualization, and Integrated Discovery (DAVID) online tool (http://david.abcc.ncifcrf.gov). GSEA was performed with the database MSigDBv7.2. NCBI Sequence Read Archive sequencing data were uploaded under accession number PRJNA1029464.

### GST pulldown and MS

HEK-293T cells with 25% confluence were plated overnight, and the medium was replaced after adherence the next day with a fresh medium containing 10% FBS. Six micrograms of p-MS2-circSKA3 or 6 μg of p-MS2 control plasmid and 4 μg of PCDH-MS2-GST-PURO-NES plasmid (containing MS2 binding protein that can recognize MS2 RNA, a GST tag can be bound by GST latex beads, and the cytoplasmic localization signal NES) were transfected into HEK-293T cells using Neofect transfection reagent for 48 h [15]. The cells were washed twice with precooled PBS, and then 800 μl of lysate (containing 20 mM Tris-HCl at pH 7.5, 100 mM KCl, 5 mM MgCl_2_, 0.5% NP-40, protease inhibitors (Beyotime, China), RNase inhibitor (Beyotime, China), and 10 mM DTT (Beyotime, China)) was added [15]. After lysis on ice for 10 min, the supernatant was collected by centrifugation at 10,000 × *g* for 10 min. GST latex beads (30 μl) were prepared for each tube, and 500 μl of lysis buffer was used for washing twice at 2000 rpm for 2 min. After centrifugation, 50 μl of supernatant was reserved as input for RNA and protein, and the rest of the supernatant was added to the washed GST latex beads, which were incubated at 4 °C on a rotating shaker for 12 h. After washing 5 times, the beads were resuspended in 500 μl of lysis buffer, and 50 μl was reserved as the IP sample of RNA. The remaining samples were centrifuged to collect the latex beads, and 60 μl of lysate diluted with 2x SDS loading buffer was added to denature the protein at 100 °C for 5 min. The proteins were separated by SDS‒PAGE, and the differential bands were identified using a silver staining kit (Sangon, CAT#C500021-0010). The bands were cut out for MS detection. The target proteins identified by MS were identified by immunoblotting of the remaining samples.

### RNA immunoprecipitation (RIP)

HEK-293T cells and HCT116 cells were plated overnight in 10 cm dishes and transfected after reaching 40% confluency. Next, 4 μg of pLCDH-ciR or circSKA3 plasmid and 6 µg of PCDH-SLUG-FLAG or 6 µg of PCDH-FUS-FLAG plasmid were added for 48 h of transfection. After washing the cells twice with precooled PBS, the cells were pipetted down or collected with a scraper and centrifuged at 1500 rpm for 5 min at 4 °C to obtain cell pellets. Then, 100 μl of the prepared lysis solution was added to each tube (1000 mM KCl, 50 mM MgCl2, 100 mM HEPES-NAOH, 5% NP-40 diluted with water; 1 mM DTT, 200 U/ml RNase inhibitor and cocktail were added when used), and the cells were placed in a −80 °C freezer to lyse overnight. After the tubes were thawed on ice, the supernatant was collected by centrifugation at 13,300 rpm for 10 min. Next, 500 μl of NET-2 was added to each 30 μl of FLAG magnetic beads, and the beads were briefly centrifuged and washed three times for later use. Then, 20 μl of supernatant was reserved as input for RNA and protein. The remaining supernatant was added to the magnetic beads, and the beads were incubated for 12 h at 4 °C with shaking rotation. The magnetic beads were washed 6 times with 500 μl of NET-2, 50 μl of the sample was reserved as the immunoprecipitation (IP) sample of protein in the last washing, and the remaining magnetic beads were used as the sample for RNA extraction. The enrichment of target RNA was identified by RT‒qPCR.

### In vitro ubiquitylation assay

HEK-293T cells were plated at 30% overnight in a 6 cm dish, and 1 μg of Ub-HA, 1 μg of SLUG-FLAG, and 1 μg of circSKA3 plasmids were transfected into the cells with lipD293. After 42 h of transfection, 20 μM MG132 was added, and the incubation was terminated after 6 h. The cells were gently washed 3 times with precooled PBS, and 1/8 of the cells were taken for RNA input in the last pipetting. Then, 600 μl of lysis buffer (RIPA+50x cocktail+100x PMSF) was added to each sample. All cells were pipetted and lysed on ice, and the cells were lysed by shaking at 5 min intervals for 30 min. After centrifugation at 13300 rpm for 10 min, 60 μl of the supernatant was removed as the protein input, and 1× SDS loading was added to denature the protein. We aspirated 20 μl of FLAG magnetic beads for each sample and washed the magnetic beads three times with TBS for use. The remaining supernatant after centrifugation was added to the magnetic beads, and the tubes were placed on a rotary shaker at 4 °C overnight. The magnetic beads were washed 3 times with TBS and resuspended in 50 μl of 2x SDS loading buffer, and then the protein was denatured for immunoblotting.

### Quantification and statistical analysis

The data in this paper are presented as the mean ± standard deviation (SD) or mean ± standard error of the mean (SEM) (from at least three biological replicates). Paired- or independent-samples Student’s *t*-tests were used to analyze the significant differences between two groups, and differences among three or more groups were analyzed using ANOVA. K–M survival analysis was performed with the log-rank test. Statistical analysis was performed using GraphPad Prism 9 (GraphPad Software, Inc., San Diego, CA, USA) and SPSS v.20.0 (SPSS Inc., Chicago, IL, USA). Differences at *p* < 0.05 were considered statistically significant and are indicated with asterisks (ns, not significant with *p* > 0.05, *p < 0.05, **p < 0.01, ***p < 0.001).

### Supplementary information

Supplemental Information

Supplemental Material of western blots

aj-checklist

## Data Availability

All data and computer code analyzed in this research are included either in this article or in the Supplemental information files, and are available from the corresponding authors upon reasonable request.
