# Supplementary material for: Specific intracellular retention of circSKA3 promotes colorectal cancer metastasis by attenuating ubiquitination and degradation of SLUG
Source: Cell Death Dis. 2023 Nov 16;14(11):750. doi: 10.1038/s41419-023-06279-w (PMC10654574; doi:10.1038/s41419-023-06279-w)
Supplement: Supplementary file 1 — Supplemental Information [file 41419_2023_6279_MOESM1_ESM.docx]

**Specific intracellular retention of circSKA3 promotes colorectal cancer metastasis by attenuating ubiquitination and degradation of SLUG**

**Authors**

Jingwen Deng^1*^, Shaoxia Liao^1*^, Chaoyi Chen^2^, Fengyan Han^1^, Siqin Lei^1^, Xuan Lai^1^, Kehong Ye^1^, Qizheng Han^1^, Fang E^1^, Chao Lu^3^, Maode Lai^4, 5^, Fanlong Liu^6 #^ and Honghe Zhang^1, 5 #^

**Affiliations**

^1^ *Department of Pathology and Women’s Hospital, Zhejiang University School of Medicine, Research Unit of Intelligence Classification of Tumor Pathology and Precision Therapy, Chinese Academy of Medical Sciences (2019RU042), Hangzhou 310058, China*

^2^ *Department of Colorectal Surgery and Oncology, Key Laboratory of Cancer Prevention and Intervention, Ministry of Education, The Second Affiliated Hospital, Zhejiang University School of Medicine, Hangzhou, Zhejiang, China*

^3^ *Department of Chemistry, University of South Florida, Tampa, Florida 33620, United States*

^4^ *Department of Pathology, Research Unit of Intelligence Classification of Tumor Pathology and Precision Therapy of Chinese Academy of Medical Sciences (2019RU042), Zhejiang University School of Medicine, Hangzhou 310058, China*

^5^ *Key Laboratory of Disease Proteomics of Zhejiang Province, Zhejiang University, Hangzhou 310058, China*

^6^ *Department of Colorectal Surgery, The First Affiliated Hospital, College of Medicine, Zhejiang University, Hangzhou 310058, China*

^*^These authors contributed equally to this work.

^#^Corresponding author E-mail: [fanlong_liu@zju.edu.cn](mailto:fanlong_liu@zju.edu.cn) (F. Liu) and [honghezhang@zju.edu.cn](mailto:honghezhang@zju.edu.cn) (H. Zhang)

# Supplemental figure legends


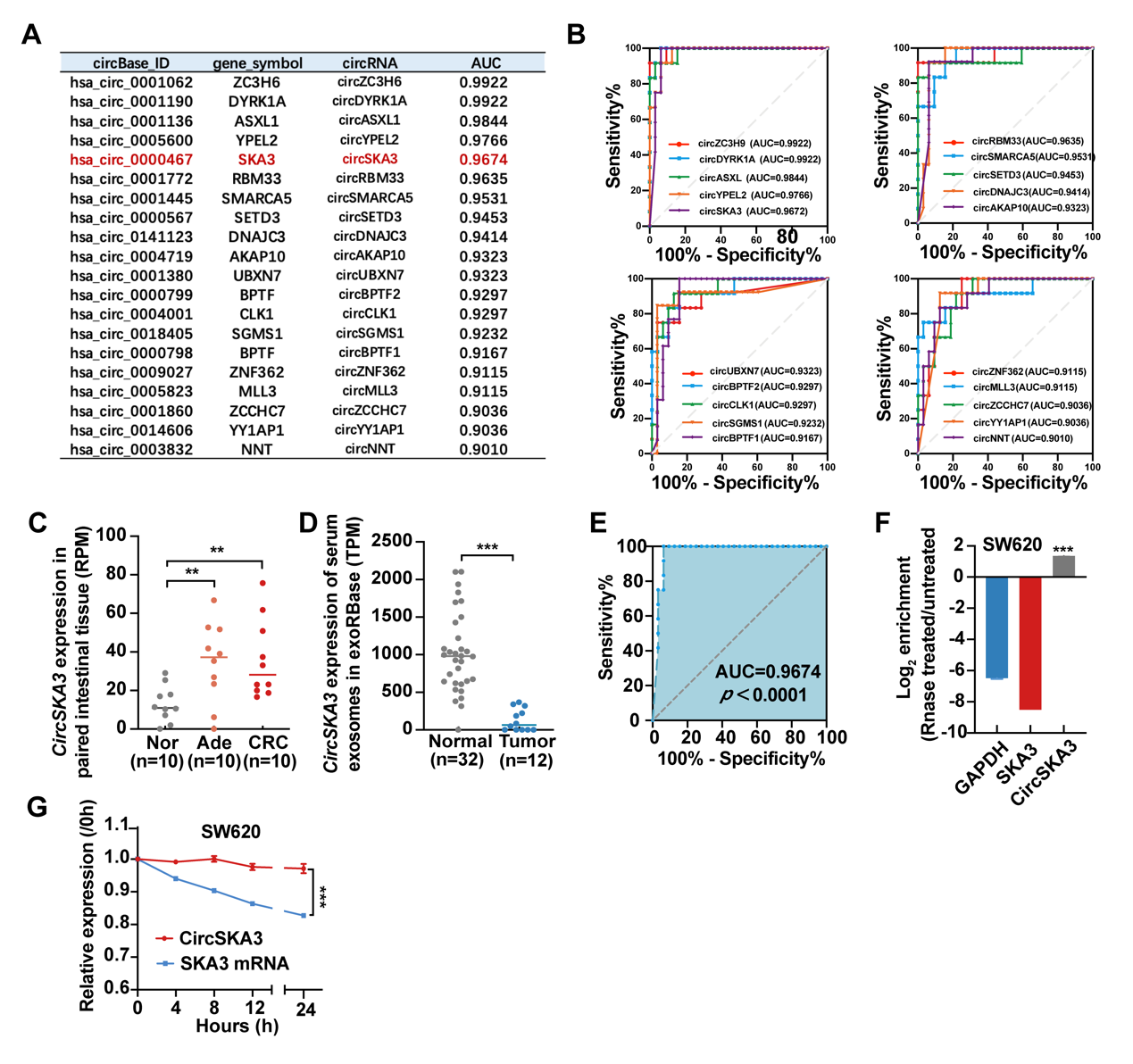


**Fig. S1 Analysis of circSKA3 expression data from the GEO database and circRNA sequencing data.** (**A and B**) Top 20 circRNAs with AUC > 0.9 from serum exosome sequencing of normal control and CRC patient data in the GEO database. (**C**) Expression of circSKA3 in 10 paired normal intestinal mucosa–adenoma/adenocarcinoma tissue samples. (**D**) Expression levels of circSKA3 in serum exosomes from normal controls and CRC patients in the GEO database. (**E**) ROC curve of circSKA3 in serum exosomes from normal control and CRC patient data in the GEO database. (**F**) The stability of circSKA3 and linear SKA3 in SW620 was assessed by RNase R treatment followed by RT‒qPCR. (**G**) Evaluation of the stability of circSKA3 and SKA3 mRNA in SW620 cells treated with actinomycin D. All results are shown as mean ± SD. Statistical significance was accessed by Student’s t-test (D, F, G), and One-way ANOVA (C). ** *p* < 0.01, *** *p* < 0.001.


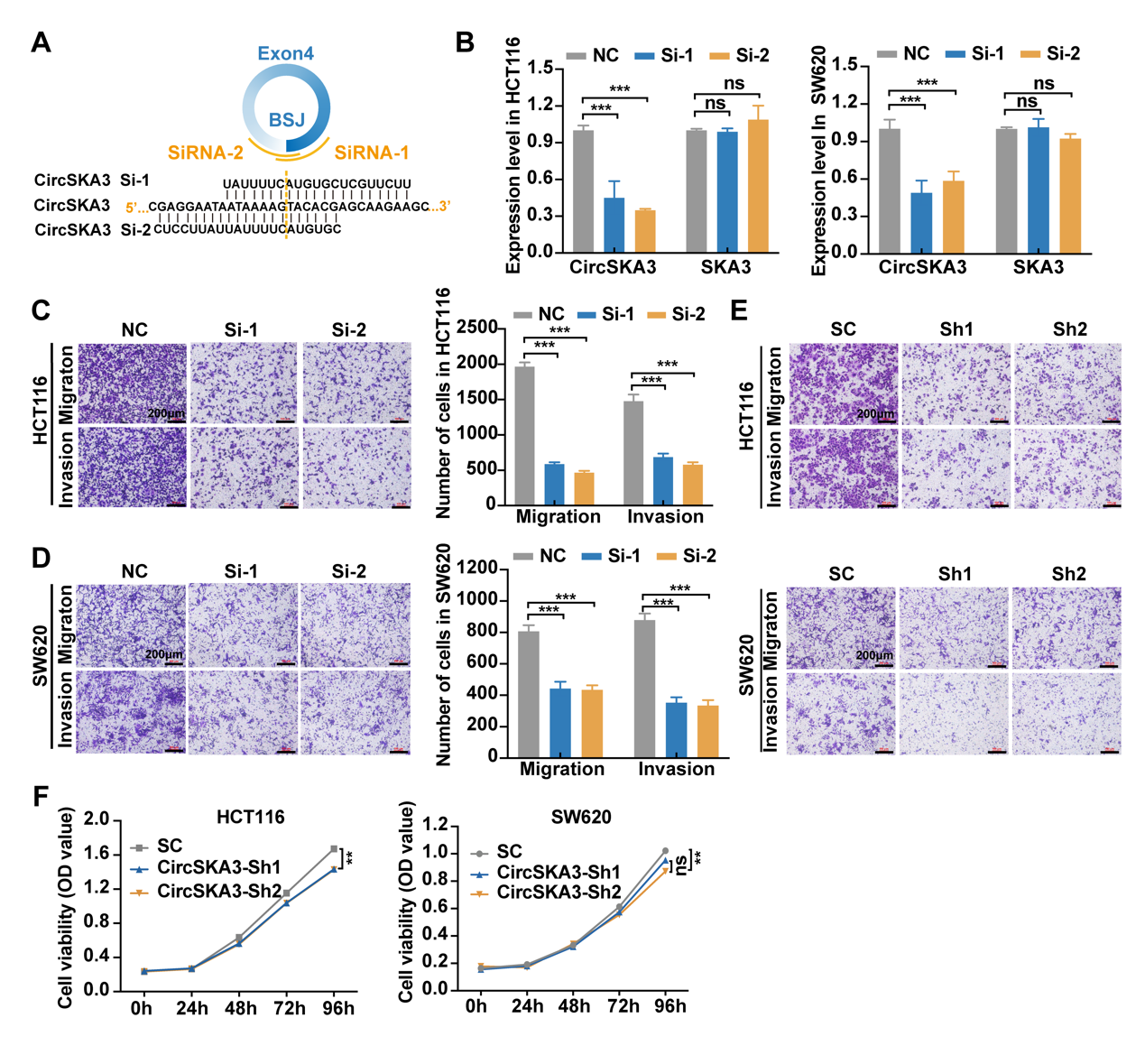


**Fig. S2 Knockdown of circSKA3 inhibits cell migration and invasion *in vitro*.** (**A**) Schematic of the siRNA sequence targeting the circSKA3 BSJ site. (**B**) Knockdown of circSKA3 via siRNA in HCT116 and SW620 cells. (**C and D**) Transwell assay for the migration and invasion abilities of HCT116 and SW620 after circSKA3 knockdown by siRNA. The right graph shows the results of quantitative analysis (scale bar=200 μm). (**E**) Transwell assay for the migration and invasion abilities of HCT116 and SW620 after shRNA-mediated circSKA3 knockdown (scale bar=200 μm). (**F**) Effect of circSKA3 knockdown via shRNA on HCT116 and SW620 cell proliferation. All results are representative of three independent experiments, and shown as mean ± SD (B, F) or mean ± SEM (C, D). Statistical significance was accessed by One-way ANOVA (B, C, D, F). ns, not significant with *p* > 0.05, ***p* < 0.01, ****p* < 0.001.


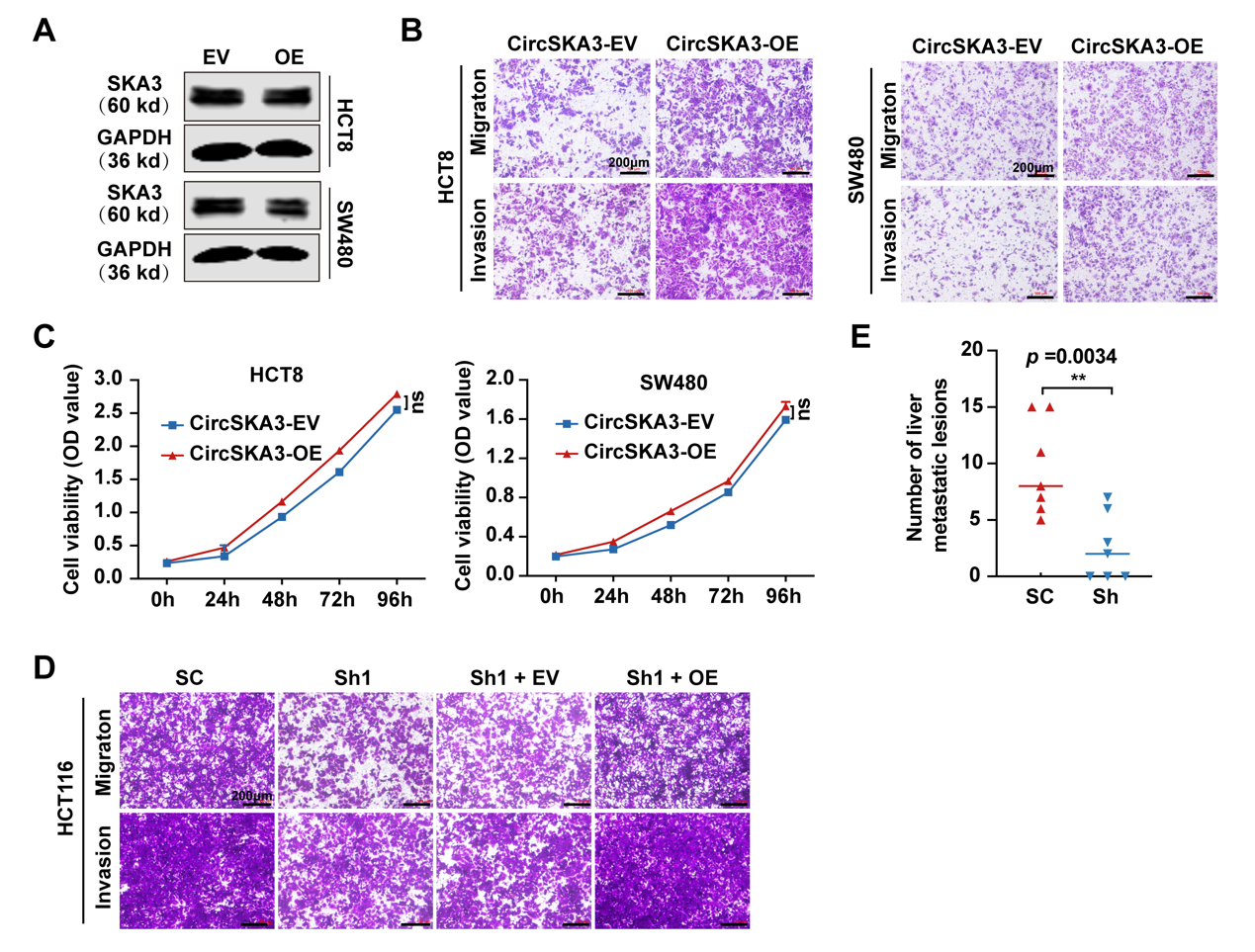


**Fig. S3 CircSKA3 promotes CRC migration and invasion *in vitro*.** (**A**) Detection of the protein level of the host gene SKA3 after circSKA3 overexpression by immunoblotting. (**B**) Transwell assay for the migration and invasion abilities of HCT8 and SW480 cells after circSKA3 overexpression (scale bar=200 μm). (**C**) Effects of circSKA3 overexpression on HCT8 and SW480 cell proliferation. (**D**) Transwell assay for the migration and invasion abilities of circSKA3-knockdown HCT116 cells (scale bar=200 μm). (**E**) Quantitative analysis of the number of liver metastatic lesions in the spleen-liver metastasis model. All results are representative of three independent experiments, and shown as mean ± SD (C, D). Statistical significance was accessed byOne-way ANOVA (C, E). ns, not significant with *p* > 0.05, ***p* < 0.01.


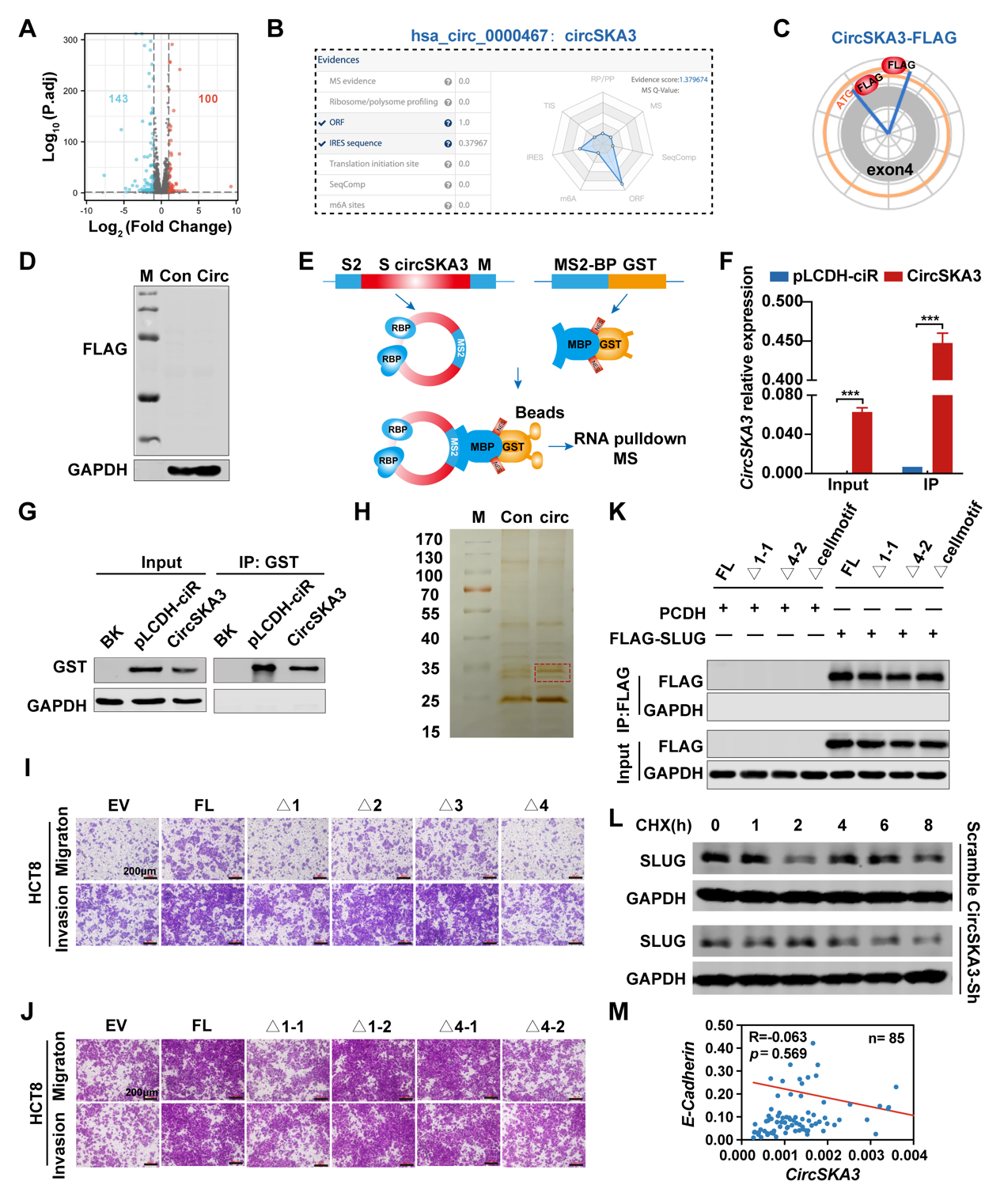


**Fig. S4 Key functional motifs of CircSKA3.** (**A**) Volcano plot of differentially expressed genes in circSKA3-knockdown cells. (**B**) Prediction of the ORF and IRES of circSKA3. (**C**) FLAG-tagged circSKA3 vector construction. (**D**) Detection of Flag in circSKA3-FLAG-OE vector-transfected 293T cells by immunoblotting. (**E**) Schematic diagram of the circSKA3 MS2-GST RNA pulldown assay. (**F**) CircSKA3 enrichment analysis for the MS2-GST RNA pulldown assay. (**G**) Detection of GST in the MS2-GST RNA pulldown assay. (**H**) SDS‒PAGE MS2-GST RNA pulldown assay. (**I and J**) Transwell assay analysis for the migration and invasion abilities of HCT8 cells transfected with different truncated circSKA3 constructs or FL circSKA3 (scale bar=200 μm). (**K**) Detection of FLAG in the RIP assay. (**L**) Detection of SLUG protein levels at different time points after CHX treatment of CRC control and knockdown HCT116 cells by immunoblotting. (**M**) Correlation between circSKA3 and E-Cadherin mRNA levels in human CRC samples. All results are representative of three independent experiments, and shown as mean ± SD (F). Statistical significance was accessed by Student’s t-test (F). ****p* < 0.001.

**Table S1. Oligonucleotide Sequences used in this study**

| **circSKA3 siRNA and shRNA** | | |
| --- | --- | --- |
| **Name** | **sense** | **antisense** |
| siRNA1 | AUAAAAGUACACGAGCAAGAA | UUCUUGCUCGUGUACUUUUAU |
| siRNA2 | GAGGAAUAAUAAAAGUACACG | CGUGUACUUUUAUUAUUCCUC |
| NC | UUCUCCGAACGUGUCACGU | ACGUGACACGUUCGGAGAA |
| shRNA1 | ATAAAAGTACACGAGCAAGAA | TTCTTGCTCGTGTACTTTTAT |
| shRNA2 | TAATAAAAGTACACGAGCAAG | CTTGCTCGTGTACTTTTATTA |
| **Crisper gRNA sequences** | | |
| 13d gRNA1 | CTTCTTGCTCGTGTACTTTTATTATTCCTC | Cas13d |
| 13d gRNA2 | AATGGCTTCTTGCTCGTGTACTTTTATTAT | Cas13d |
| 13d gRNA3 | TGCTCGTGTACTTTTATTATTCCTCGCATT | Cas13d |
| control | GAAGAACGAGCACATCTTTTATTATTCCTC | Cas13d |
| direct repeat | gaaacaccgaacccctaccaactggtcggggtttgaaac | Cas13d-Direct repeat |
| **FUS siRNA** | | |
| **Name** | **sense** | **antisense** |
| siRNA1 | CAGAGUUACAGUGGUUAUATT | UAUAACCACUGUAACUCUGTT |
| siRNA2 | GCCAAGAUCAAUCCUCCAUTT | AUGGAGGAUUGAUCUUGGCTT |
| NC | UUCUCCGAACGUGUCACGUTT | ACGUGACACGUUCGGAGAATT |
| **qPCR primer sequences** | | |
| **Target** | **F** | **R** |
| circSKA3con | AGAAGCCATTAACTCTGACCCA | ACGATGTACCGCTCAAGTCC |
| circSKA3di | AGCCCGTAATTGTAACCCCA | TGGGTCAGAGTTAATGGCTTCT |
| circSKA3flag | AGCCCGTAATTGTAACCCCA | TTGTCATCGTCGTCCTTGTAA |
| SKA3 | AAAGCAGTGCCACCCAGTAA | TCCAGCTCGGCTTTCATCTC |
| GAPDH | GAAGGTGAAGGTCGGAGTC | GAAGATGGTGATGGGATTTC |
| β-ACTIN | GTCATTCCAAATATGAGATGCGT | GCTATCACCTCCCCTGTGTG |
| copGFP | GTGATCGGCGACTTCAAGGT | TGCTGCGGATGATCTTGTCG |
| MALAT1 | TGAGGTGTTTGATGACCCGT | AACAACTCGCATCACCGGAA |
| NEAT1 | AAACGCTGGGAGGGTACAAG | ATGCCCAAACTAGACCTGCC |
| VIMENTIN | TCACCTGTGAAGTGGATGCC | ACGAAGGTGACGAGCCATTT |
| E-CADHERIN | GCTGGACCGAGAGAGTTTCC | CGACGTTAGCCTCGTTCTCA |
| FUS | CAAAGCTATGGGGCCTACCC | TAGCCTGTGTTCTGGCTCTG |
| **ASO** | | |
| ASO1-1-1 | cctcctagTACACGAGCAAG | |
| ASO1-1-2 | GAATAATAAAAGgtaagcag | |
| ASO4-2-1 | CTAAATTAGAACACTTTGGT | |
| ASO4-2-2 | ATACTATGTGTTTAAATGAA | |
| NC | GCGUATTATAGCCGAUUAAC | |

**Table S2. Vectors used in this study**

| **Vector name** | **Vector** | **Application** |
| --- | --- | --- |
| circSKA3-OE | pLCDH-ciR | Overexpression |
| circSKA3-flag-OE | pLCDH-ciR | Overexpression |
| circSKA3Δ1 | pLCDH-ciR | Overexpression |
| circSKA3Δ2 | pLCDH-ciR | Overexpression |
| circSKA3Δ3 | pLCDH-ciR | Overexpression |
| circSKA3Δ4 | pLCDH-ciR | Overexpression |
| circSKA3Δ1-1 | pLCDH-ciR | Overexpression |
| circSKA3Δ1-2 | pLCDH-ciR | Overexpression |
| circSKA3Δ2-1 | pLCDH-ciR | Overexpression |
| circSKA3Δ2-2 | pLCDH-ciR | Overexpression |
| circSKA3Δ4-1 | pLCDH-ciR | Overexpression |
| circSKA3Δ4-2 | pLCDH-ciR | Overexpression |
| circSKA3Δcellmotif | pLCDH-ciR | Overexpression |
| Scramble | pLKO.1-TRC | shRNA coltrol |
| BSJ-DR-control | pLKO.1-TRC | Control |
| BSJ-DR-gRNA-1 | pLKO.1-TRC | CRISPR/Cas13d |
| BSJ-DR-gRNA-2 | pLKO.1-TRC | CRISPR/Cas13d |
| BSJ-DR-gRNA-3 | pLKO.1-TRC | CRISPR/Cas13d |
| RfxCas13d | p23-NLS-RfxCas13d-mcherry-NLS-Flag | CRISPR/Cas13d |
| MS2-GST-PURO-NES | PCDH | RNA pulldown |
| MS2-circSKA3 | pLCDH-ciR | RNA pulldown |
| FLAG-SLUG-OE | PCDH | RIP |
| FLAG-FUS-OE | PCDH | RIP |
