# Supplementary material for: Specific intracellular retention of circSKA3 promotes colorectal cancer metastasis by attenuating ubiquitination and degradation of SLUG
Source: Cell Death Dis. 2023 Nov 16;14(11):750. doi: 10.1038/s41419-023-06279-w (PMC10654574; doi:10.1038/s41419-023-06279-w)
Supplement: Supplementary file 2 — Supplemental Material of western blots [file 41419_2023_6279_MOESM2_ESM.pptx]

## Slide 1
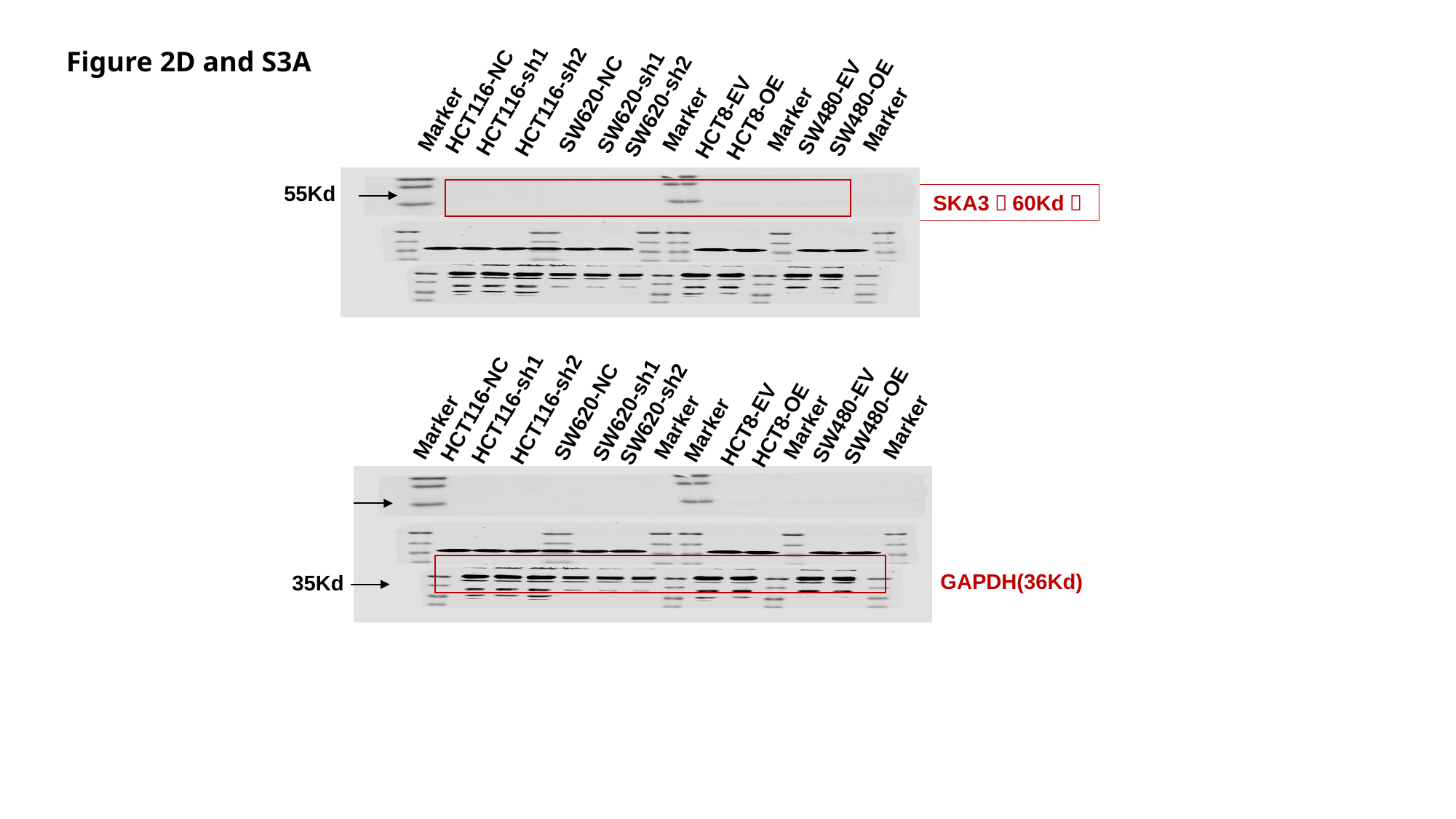

Figure 2D and S3A
HCT116-NC
HCT116-sh1
HCT116-sh2
SW620-sh1
SW620-NC
SW620-sh2
SW480-EV
SW480-OE
HCT8-EV
HCT8-OE
Marker
Marker
Marker
Marker
55Kd
SKA3（60Kd）
HCT116-NC
HCT116-sh1
HCT116-sh2
SW620-sh1
SW620-NC
SW620-sh2
SW480-EV
SW480-OE
HCT8-EV
HCT8-OE
Marker
Marker
Marker
Marker
Marker
GAPDH(36Kd)
35Kd

## Slide 2
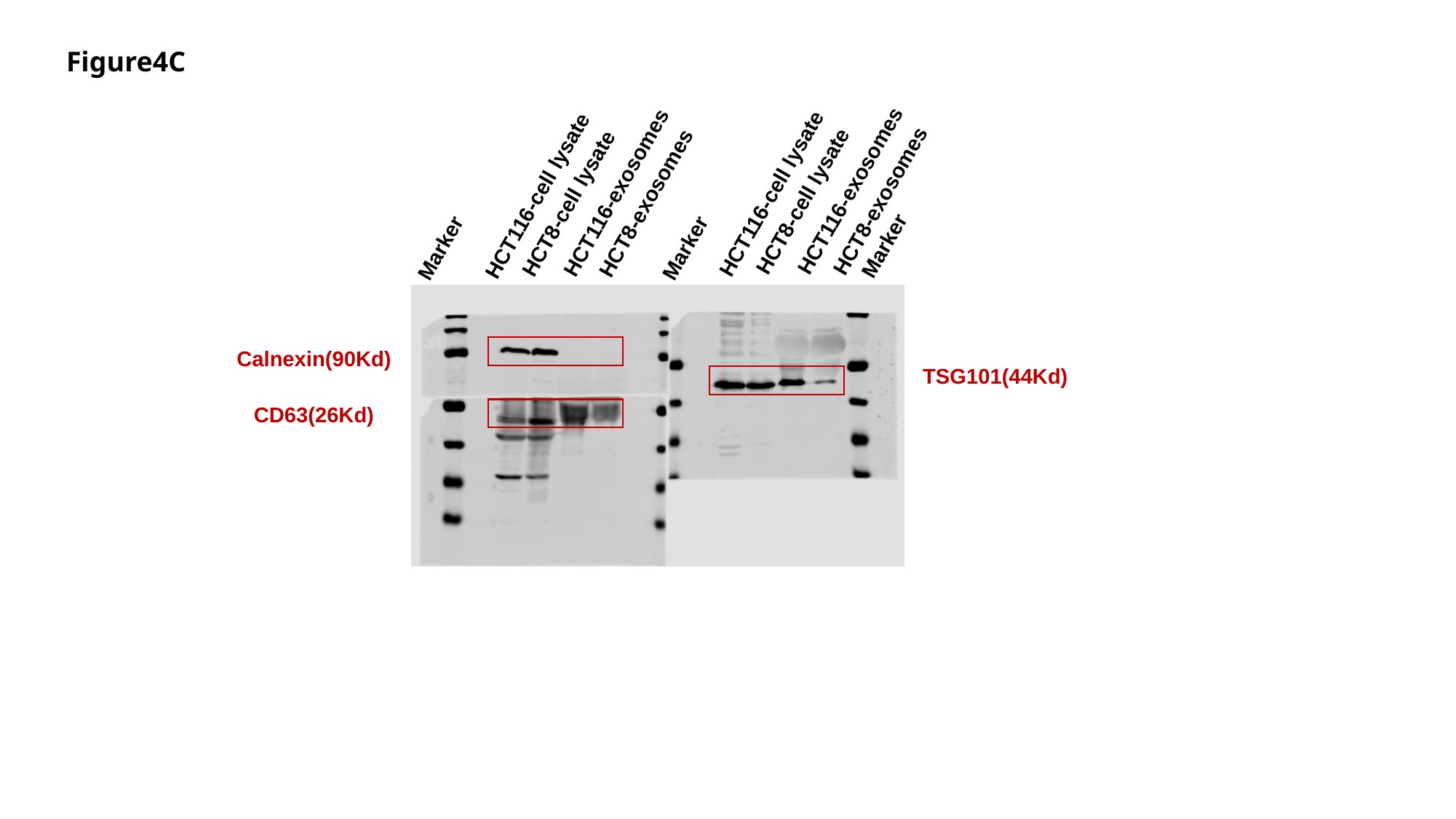

Figure4C
HCT116-exosomes
HCT116-exosomes
HCT116-cell lysate
HCT116-cell lysate
HCT8-exosomes
HCT8-cell lysate
HCT8-exosomes
HCT8-cell lysate
Marker
Marker
Marker
Calnexin(90Kd)
TSG101(44Kd)
CD63(26Kd)

## Slide 3
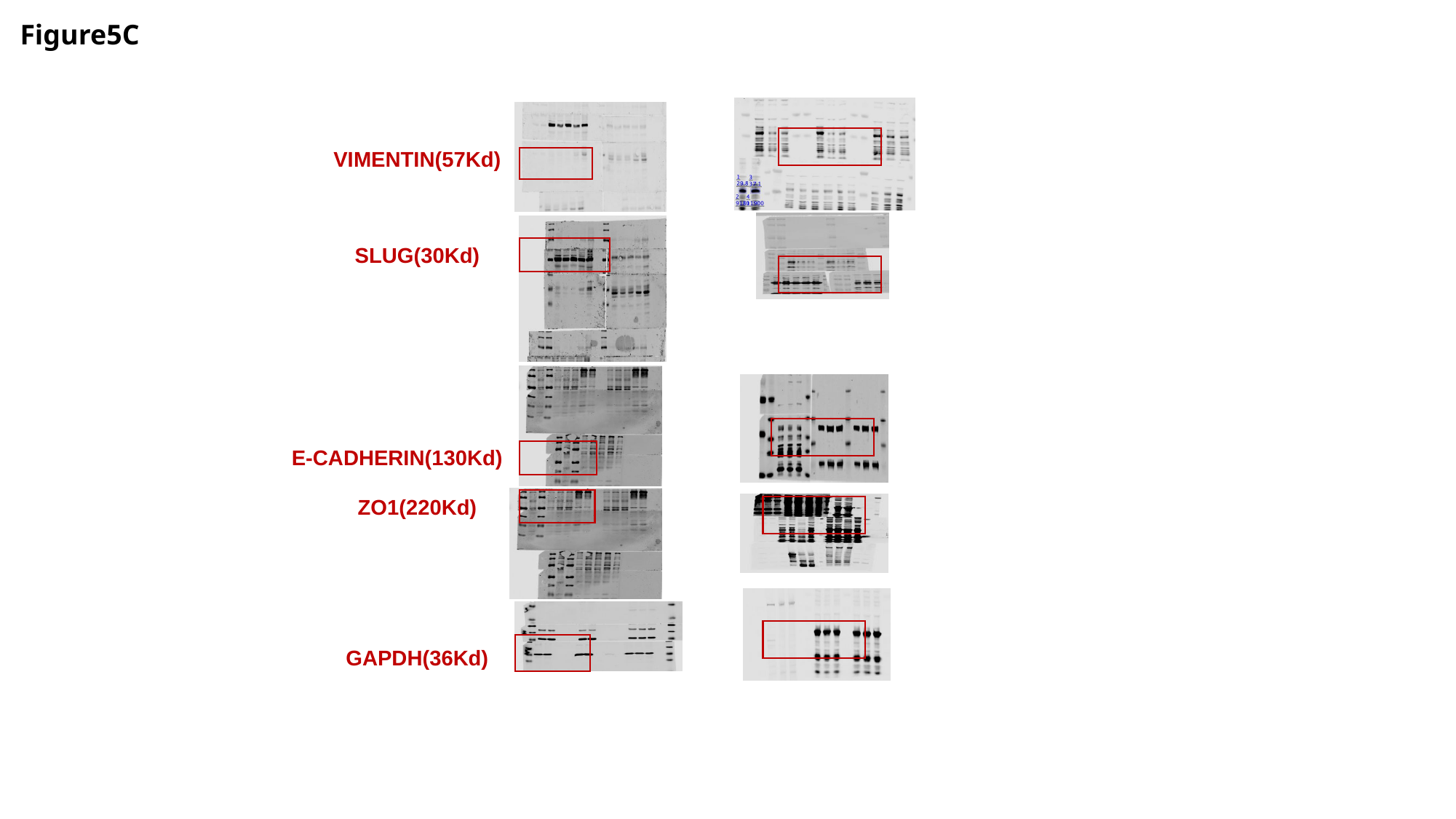

Figure5C
VIMENTIN(57Kd)
SLUG(30Kd)
E-CADHERIN(130Kd)
ZO1(220Kd)
GAPDH(36Kd)

## Slide 4
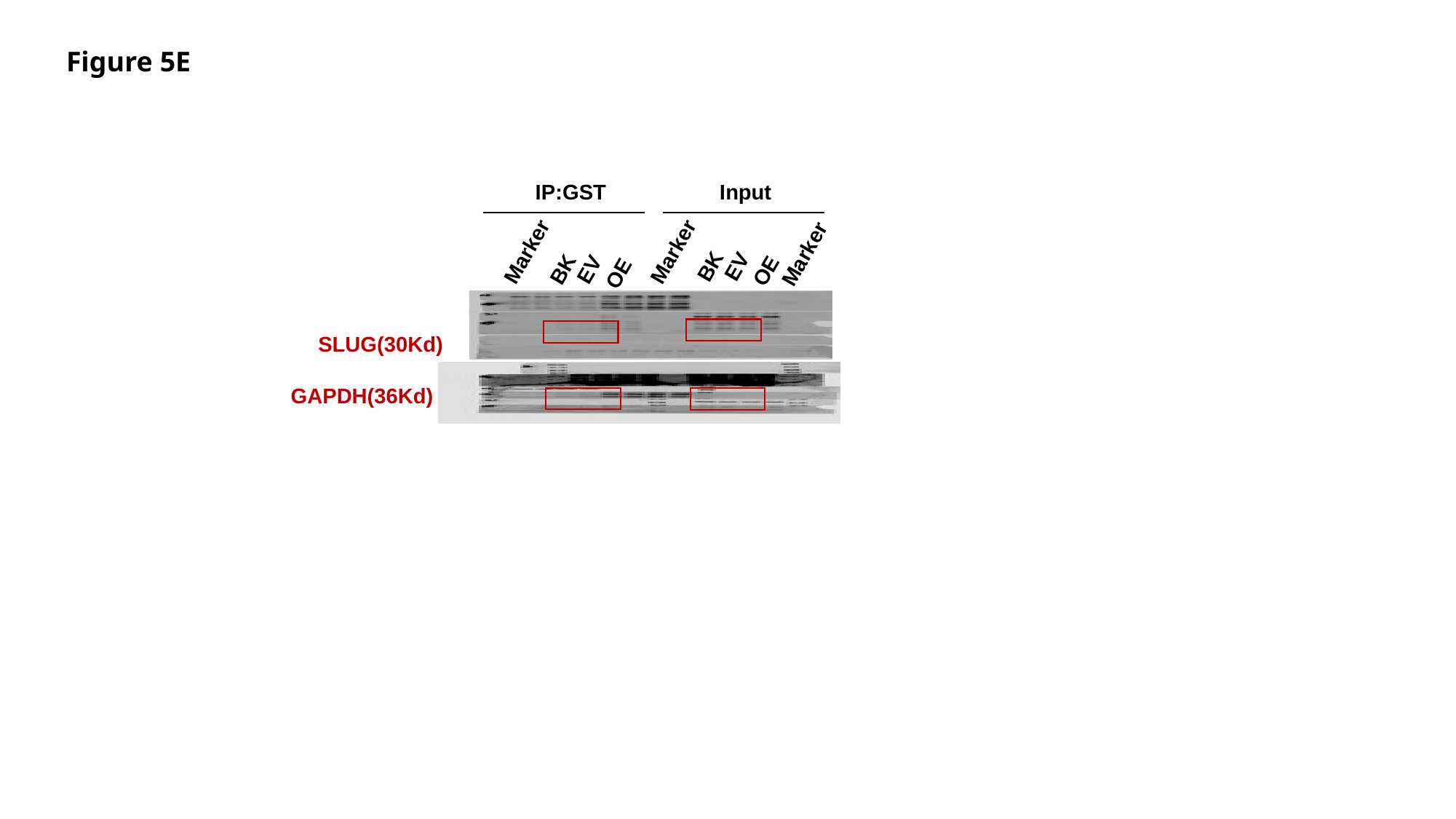

Figure 5E
Marker
IP:GST
Input
Marker
Marker
BK
EV
BK
EV
OE
OE
SLUG(30Kd)
GAPDH(36Kd)

## Slide 5
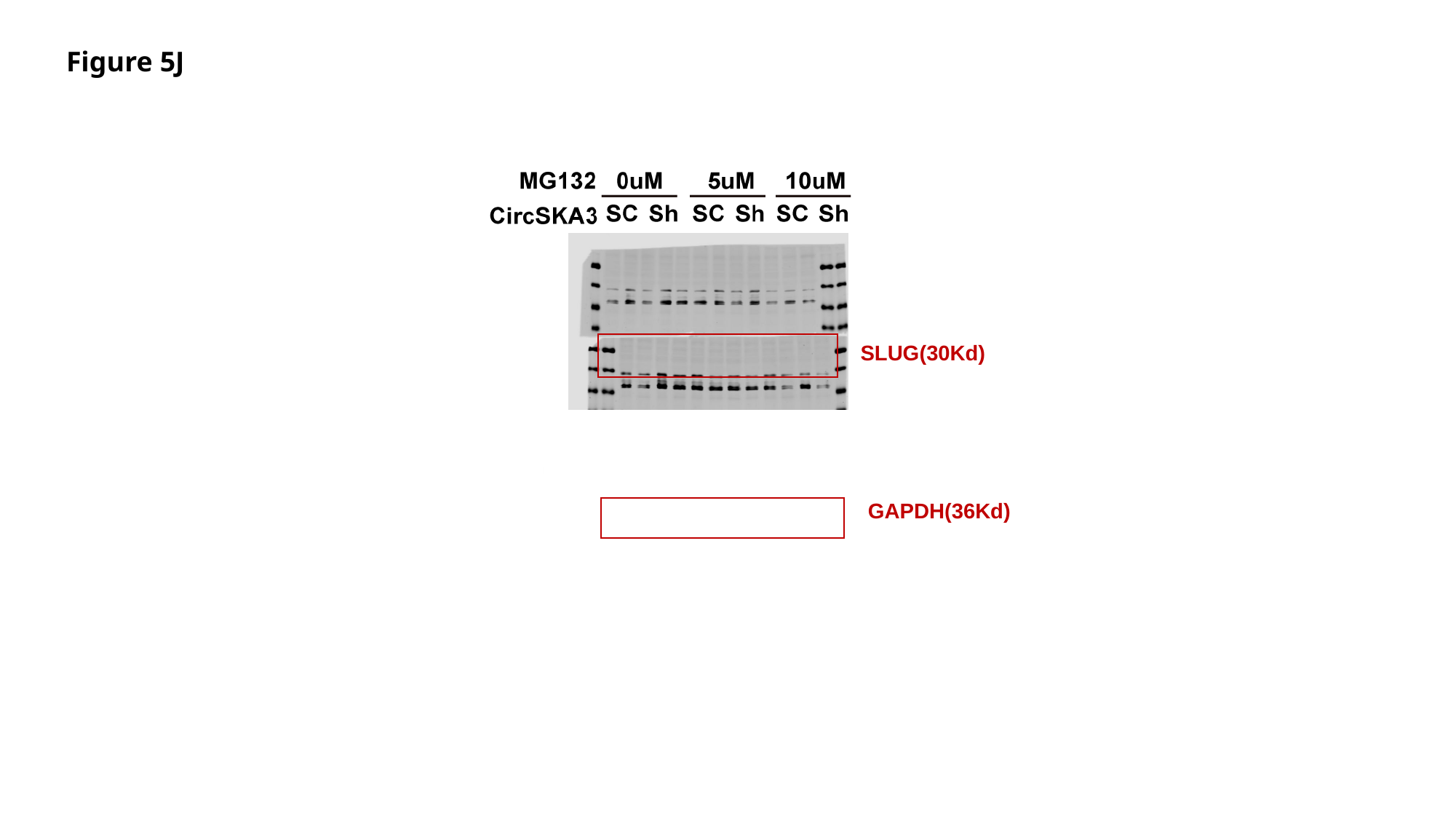

Figure 5J
SLUG(30Kd)
GAPDH(36Kd)

## Slide 6
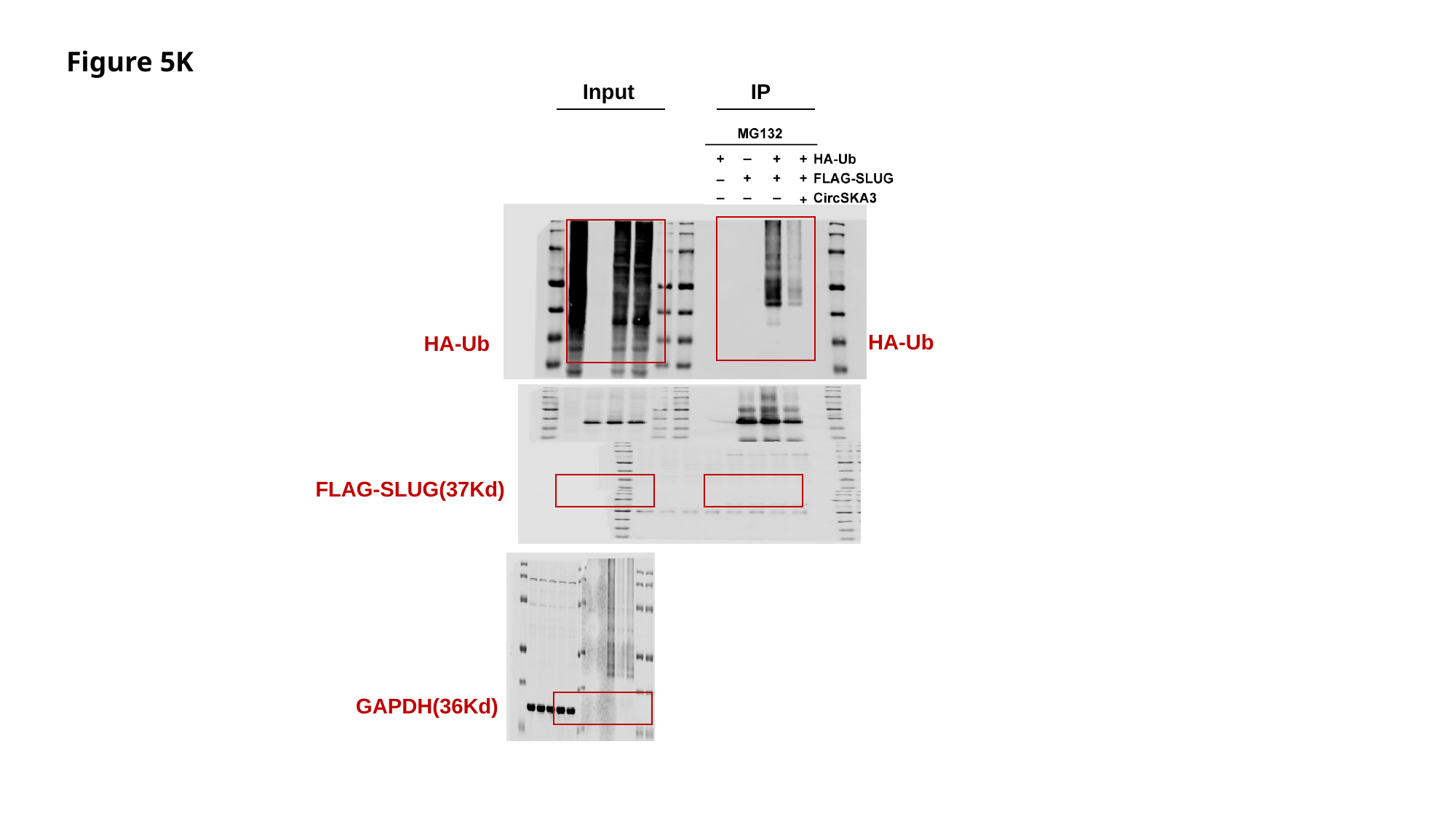

Figure 5K
Input
IP
HA-Ub
HA-Ub
FLAG-SLUG(37Kd)
GAPDH(36Kd)

## Slide 7
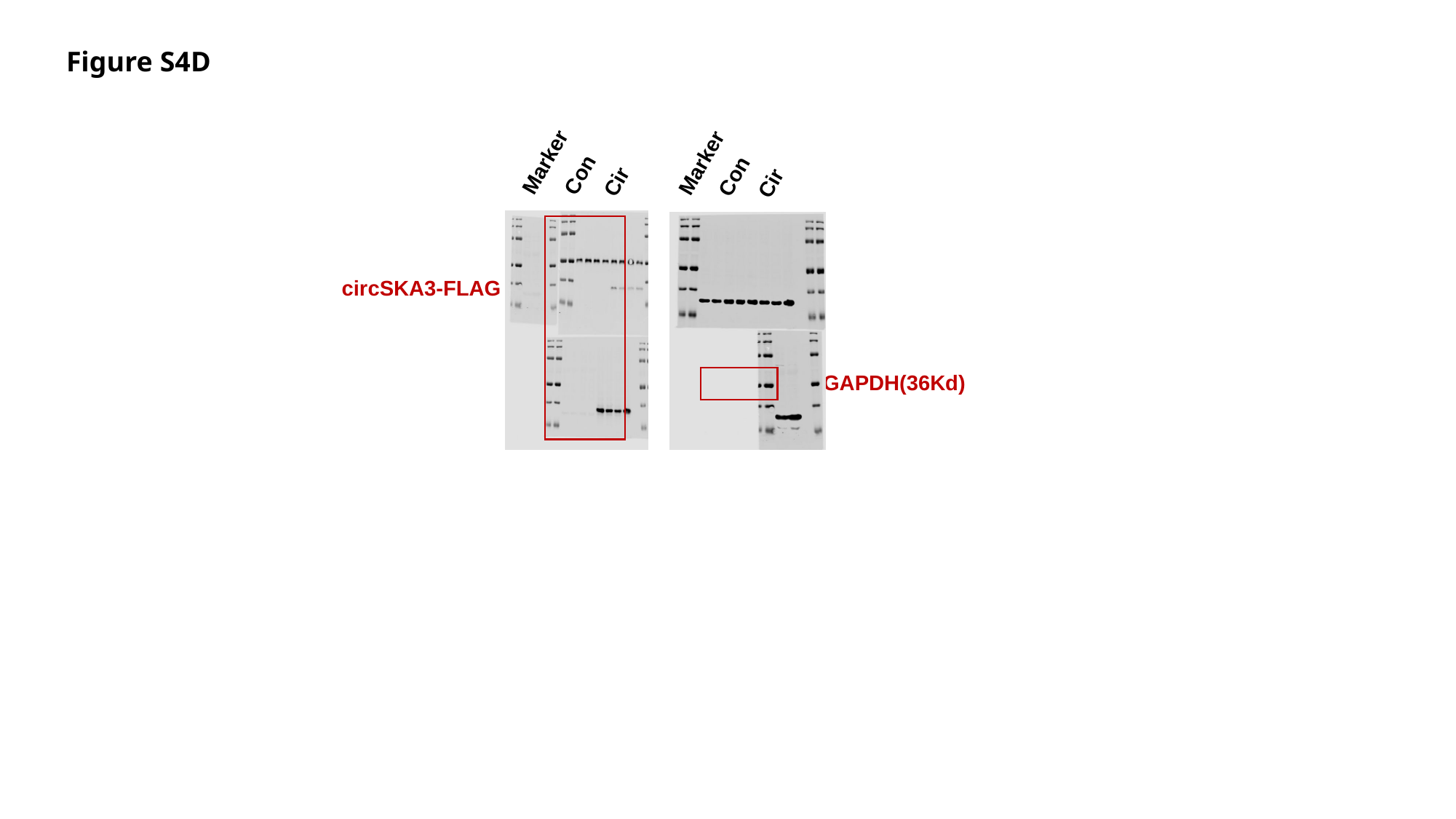

Figure S4D
Con
Con
Marker
Cir
Marker
Cir
circSKA3-FLAG
GAPDH(36Kd)

## Slide 8
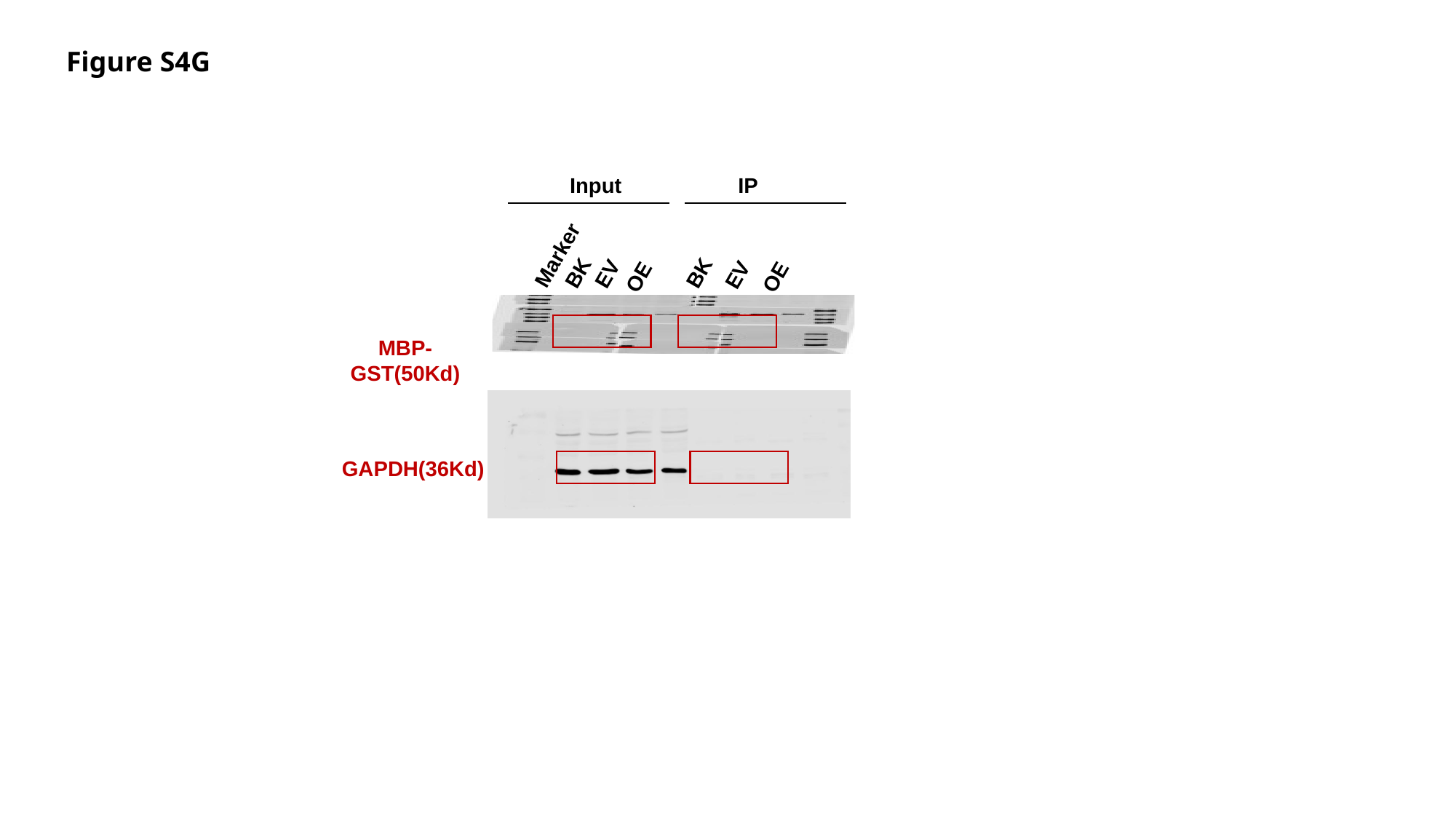

Figure S4G
Input
IP
Marker
EV
BK
BK
EV
OE
OE
MBP-GST(50Kd)
GAPDH(36Kd)

## Slide 9
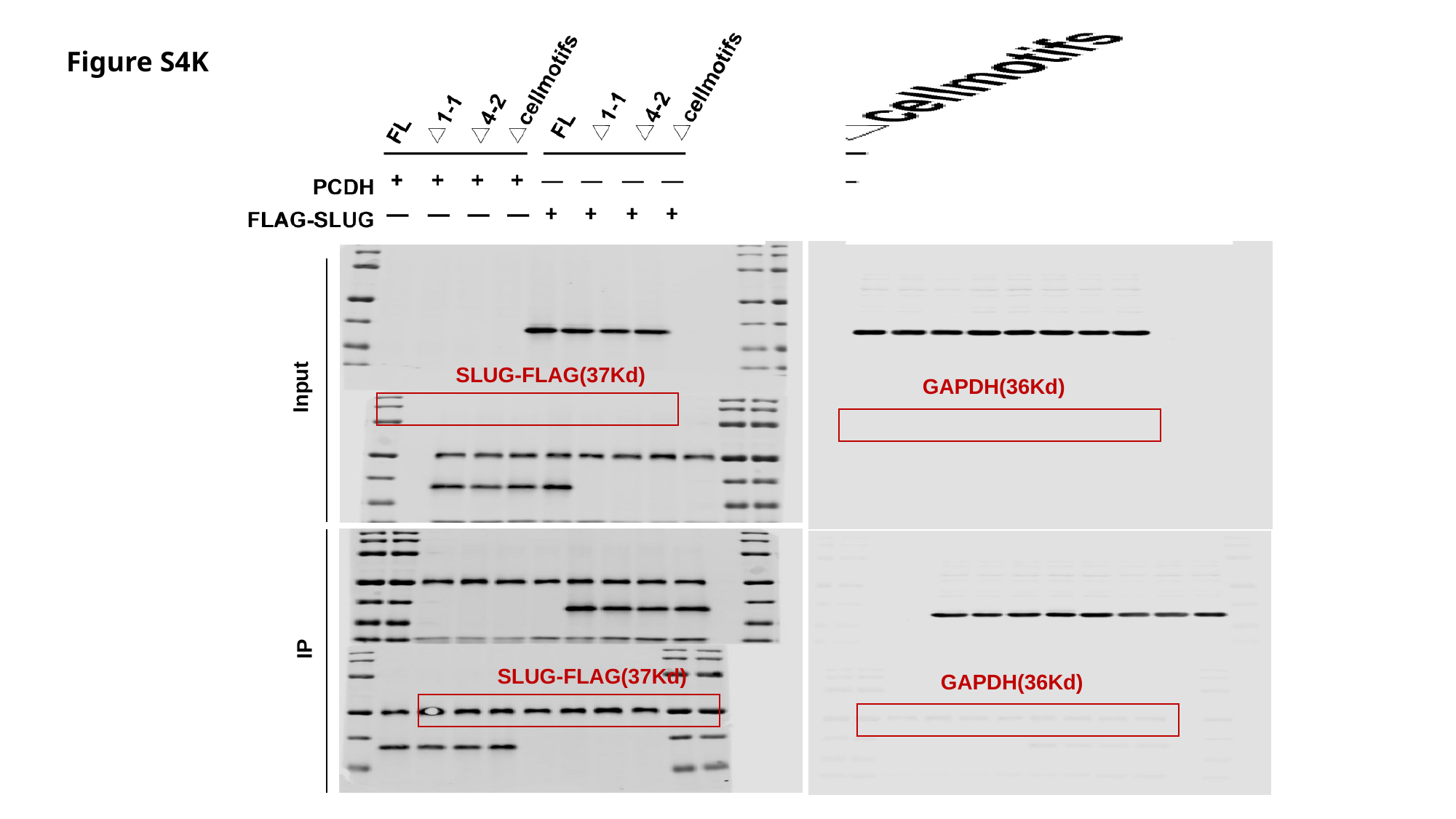

Figure S4K
SLUG-FLAG(37Kd)
GAPDH(36Kd)
Input
IP
SLUG-FLAG(37Kd)
GAPDH(36Kd)

## Slide 10
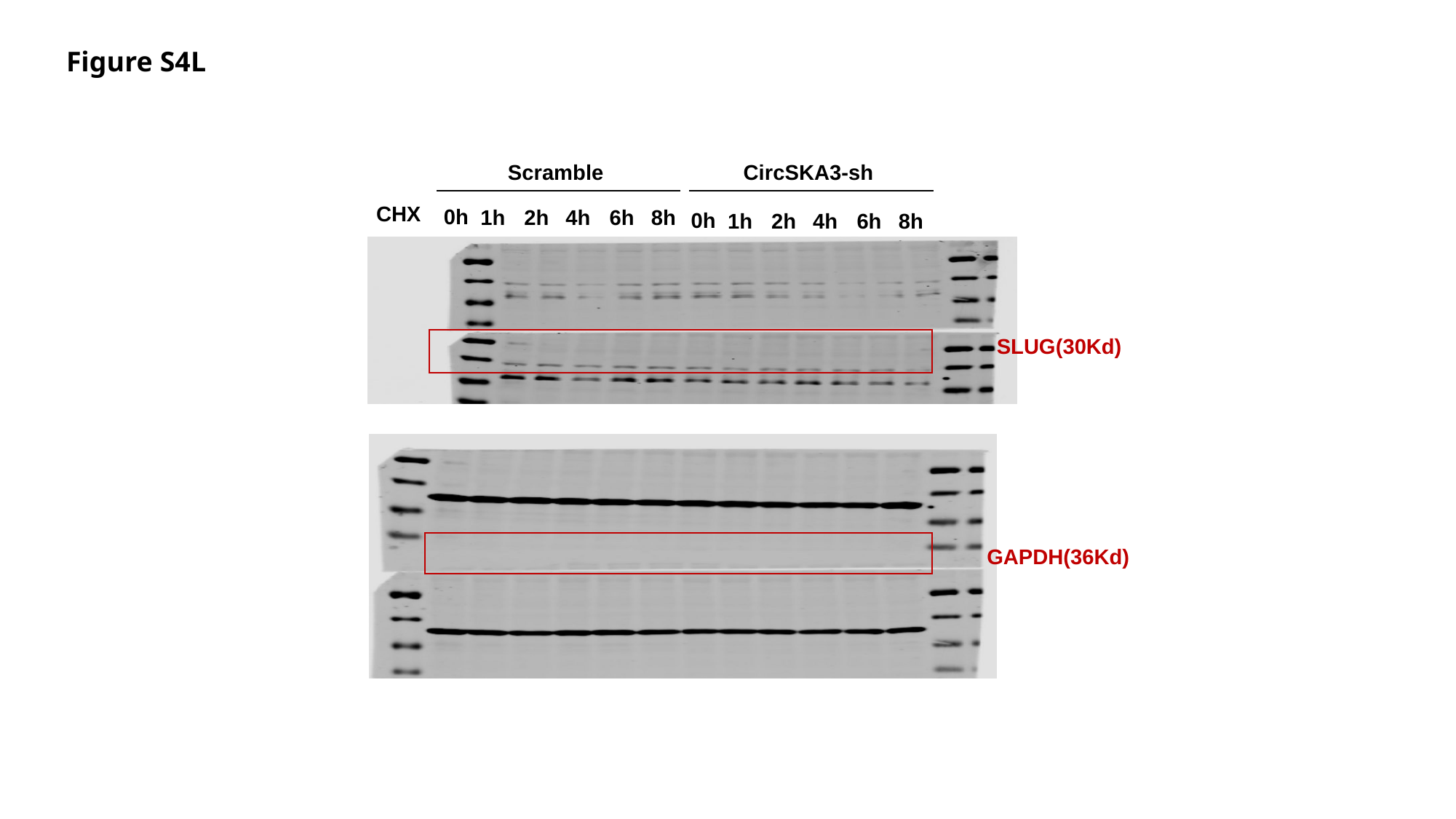

Figure S4L
Scramble
CircSKA3-sh
CHX
0h
6h
8h
1h
4h
2h
0h
6h
8h
1h
4h
2h
SLUG(30Kd)
GAPDH(36Kd)

## Slide 11
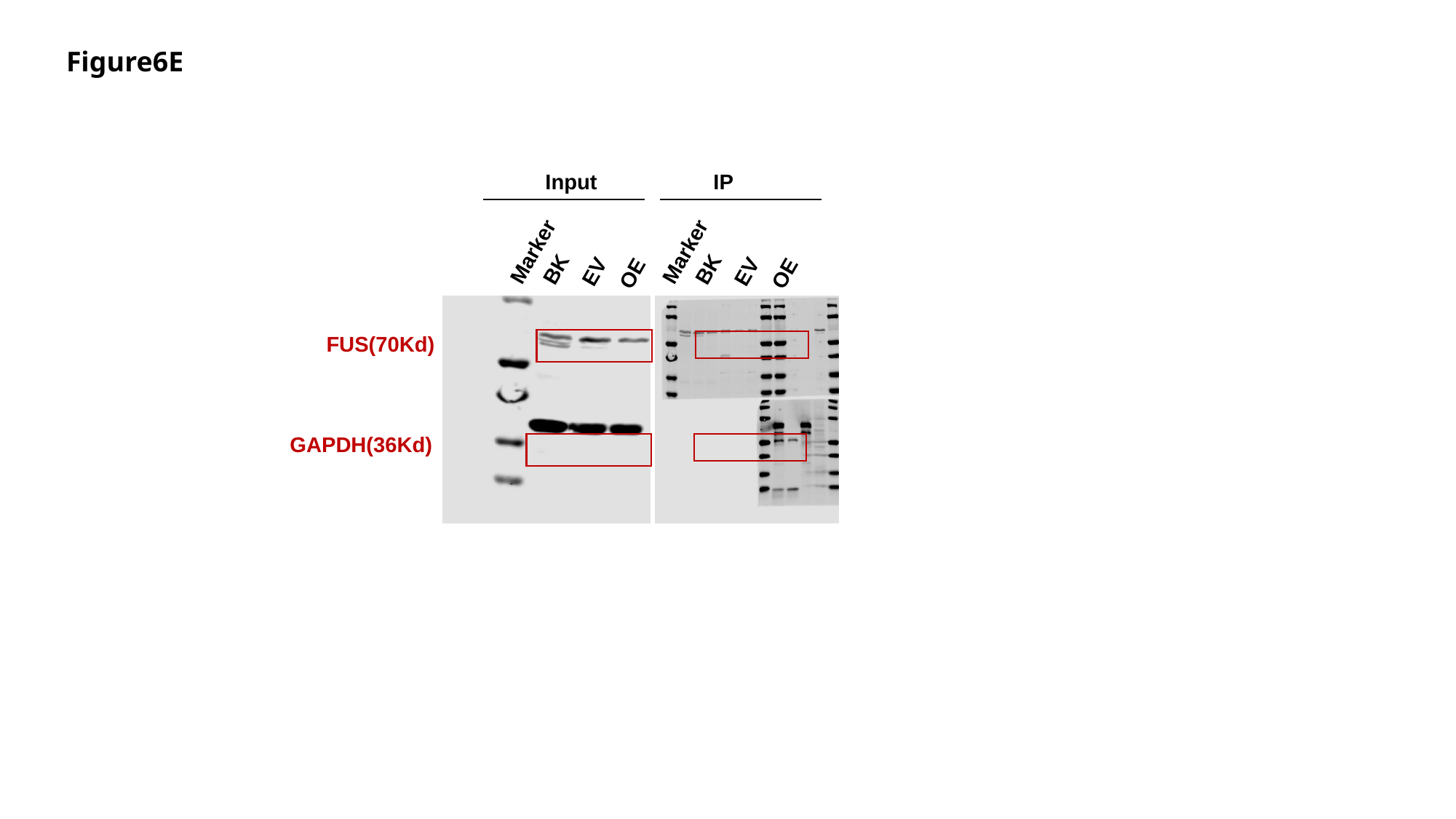

Figure6E
Input
IP
Marker
Marker
BK
BK
EV
EV
OE
OE
FUS(70Kd)
GAPDH(36Kd)

## Slide 12
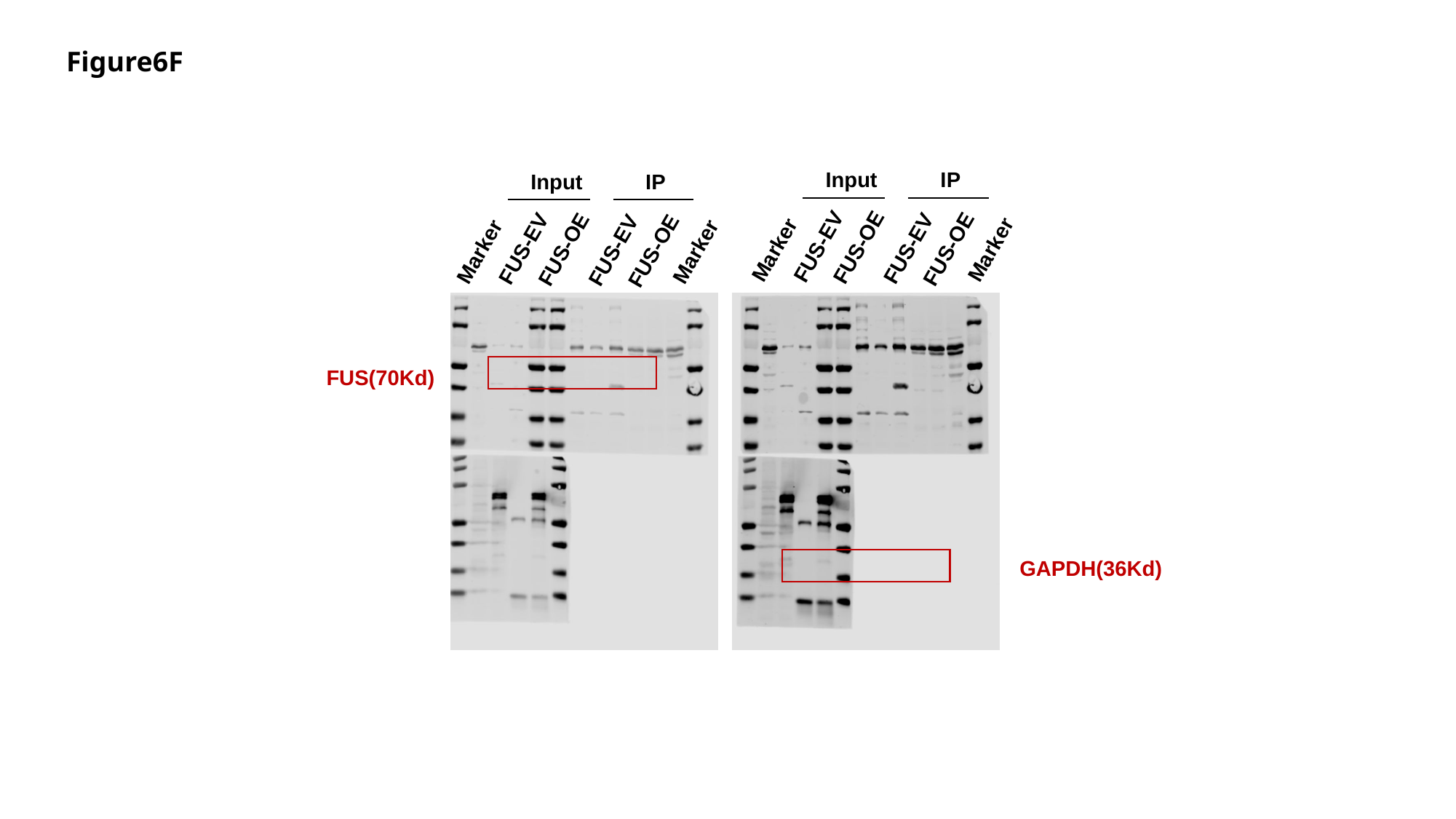

Figure6F
Input
IP
Input
IP
FUS-EV
FUS-EV
FUS-EV
FUS-EV
Marker
FUS-OE
Marker
FUS-OE
Marker
FUS-OE
FUS-OE
Marker
FUS(70Kd)
GAPDH(36Kd)

## Slide 13
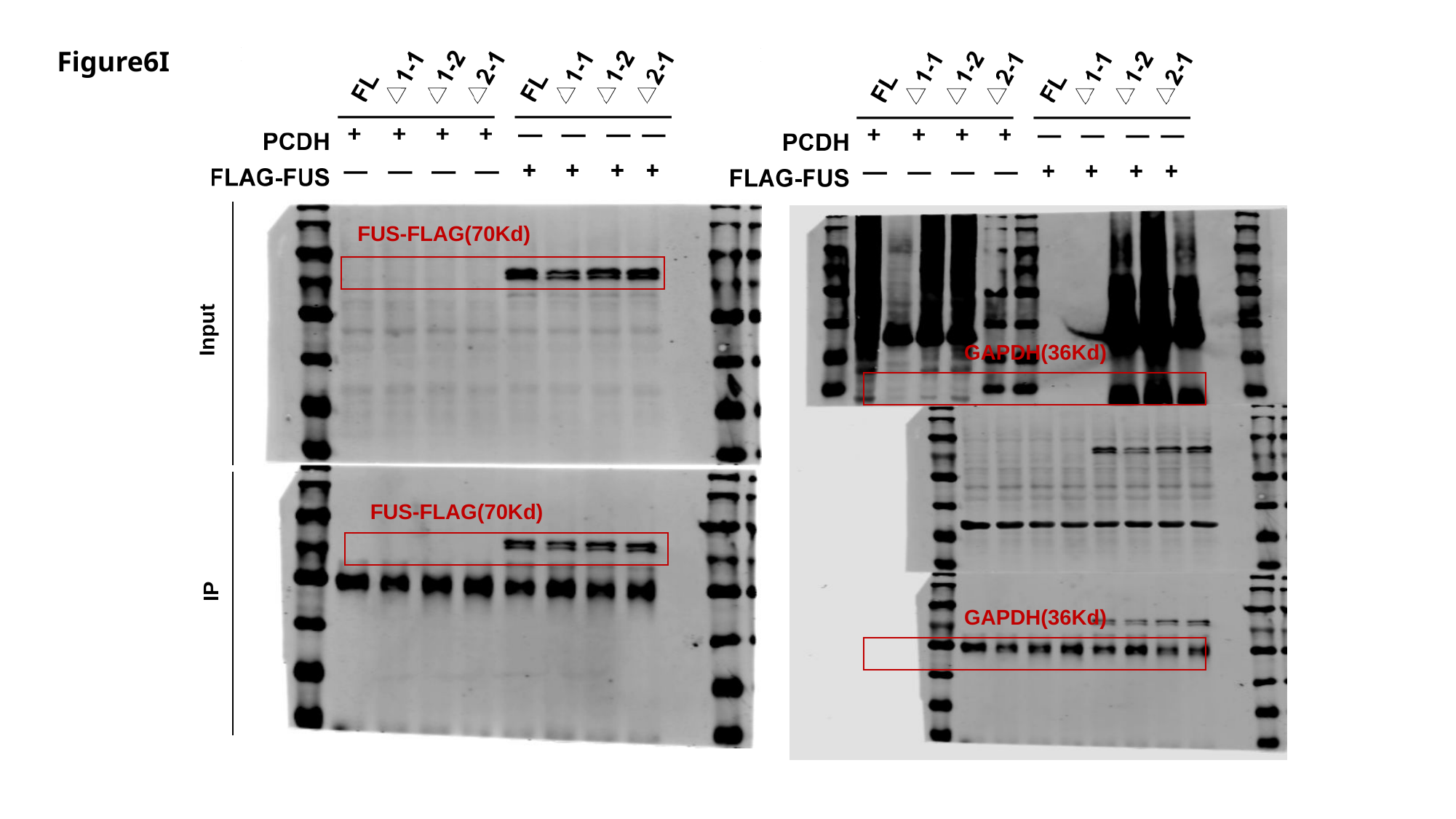

Figure6I
FUS-FLAG(70Kd)
Input
GAPDH(36Kd)
FUS-FLAG(70Kd)
IP
GAPDH(36Kd)
